# Prevalence and Associated Factors of Frailty in Patients with Chronic Heart Failure: A Systematic Review and Meta-Analysis

**DOI:** 10.31083/RCM26854

**Published:** 2025-03-24

**Authors:** Longren Wu, Si Liu, Meijun Zhang, Xiaoyun Xiong

**Affiliations:** ^1^Intensive Care Medicine, The First Affiliated Hospital of Nanchang University, Jiangxi Medical College, Nanchang University, 330006 Nanchang, Jiangxi, China; ^2^School of Nursing, Jiangxi Medical College, Nanchang University, 330006 Nanchang, Jiangxi, China; ^3^Department of Nursing, The Second Affiliated Hospital of Nanchang University, Jiangxi Medical College, Nanchang University, 330006 Nanchang, Jiangxi, China

**Keywords:** heart failure, frailty, associated factors, meta-analysis

## Abstract

**Background:**

Although numerous studies have investigated the prevalence of chronic heart failure (CHF) and the factors influencing frailty in patients with CHF, the findings remain inconsistent. Therefore, this review aimed to systematically evaluate the prevalence and associated frailty factors in patients with CHF to establish an evidence-based foundation for risk assessment and treatment strategies.

**Methods:**

A comprehensive search was conducted across multiple databases, including EMBASE, the Cochrane Library, PubMed, Web of Science, CINAHL, Chinese Biological Medicine (CBM), CNKI, and Wan Fang up to August 25, 2024. The objective was to identify observational studies that examined factors influencing frailty in CHF patients. The quality of the selected studies was evaluated using appropriate assessment tools, and a meta-analysis was performed to determine the relevant factors associated with frailty in this population.

**Results:**

A total of 23 articles containing 6287 patients were included. The prevalence of frailty in patients with CHF was 39% (95% confidence interval (CI): 0.33–0.45). Factors shown to be positively associated with frailty in CHF patients were older age, cerebrovascular accidents, longer hospital stay, larger left atrial diameter, higher number of comorbidities, poor New York Heart Association (NYHA) functional class, and poor sleep quality. Conversely, higher albumin, hemoglobin, and left ventricular ejection fraction (LVEF) levels were negatively associated with frailty.

**Conclusions:**

The prevalence of frailty in patients with CHF is relatively high and varies according to different assessment tools applied. Thus, establishing specific frailty assessment tools for CHF patients and providing targeted interventions based on important factors are essential for reducing the burden of frailty and improving outcomes.

**The PROSPERO registration:**

CRD42023448771, https://www.crd.york.ac.uk/PROSPERO/view/CRD42023448771.

## 1. Introduction

Heart failure (HF) is a multifaceted clinical condition characterized by a substantial reduction in cardiac output; HF is also often the endpoint in various cardiovascular diseases [1]. Similar to other individuals with chronic illnesses, patients with HF frequently experience multiple comorbidities, complicated medication regimens, and limited self-management abilities. Despite advances in the management of HF, the incidence, mortality, and early re-admission rates associated with HF patients remain high. Previous reports have indicated that around 64.3 million individuals are affected by HF globally [2], with re-admission rates as high as 50% within six months post-discharge. Furthermore, the 5-year survival rate for patients diagnosed with HF is only 20% [3]. It is widely recognized that rehospitalization places a considerable strain on both the healthcare system and patients, and recurrent hospitalization due to chronic heart failure (CHF) is a notable epidemiological feature of HF [4].

Frailty is a clinical state in which patients have increased vulnerability to stressors due to the decline in function and reserves of multiple physiologic systems [5]. Several assessment tools are currently available to evaluate the frailty of patients with CHF, including the frailty phenotype, the FRAIL scale, and the Tilburg Frailty Indicator scale. The frailty phenotype emphasizes the clinical manifestations of patients and primarily evaluates their frailty level across five dimensions: unintentional weight loss, self-reported exhaustion, weakness (grip strength), slow walking speed, and low physical activity [6]. The FRAIL scale is a straightforward assessment tool designed to quickly identify individuals at high risk of frailty by examining fatigue, resistance, ambulation, illnesses, and weight loss [7]. In contrast, the Tilburg Frailty Indicator is a more comprehensive evaluation that provides a multidimensional perspective on frailty status by incorporating physiological, psychological, and social factors [8]. These assessment tools enhance our understanding of frailty but vary in terms of target population, evaluation dimensions, time requirement, and implementation complexity. A recent systematic review found that frailty upon hospital admission was associated with an extended length of stay, deterioration in functional status, and increased mortality rate [9]. The prevalence of frailty in CHF is high, and frail patients with CHF face elevated risks of falls, disabilities, arrhythmias, and even death. Moreover, the addition of frailty to CHF hinders patients from understanding the required self-management skills delivered by health professionals [10, 11, 12]. Frailty poses a severe threat to the outcome of CHF patients, thus making it crucial to detect frailty and its associated risk factors early to ensure that timely interventions are delivered to prevent or cease its progression.

An increasing number of studies have explored the relationship between CHF and frailty. However, due to differences in study design, assessment tools, geographic regions, and population characteristics, a consensus on the prevalence of frailty in CHF patients and the associated risk factors remains required. The reported prevalence has ranged from 36.2% to 76.0%, depending on the intrinsic features of the study population and the tools utilized for frailty assessment [10, 13]. Some studies found that body mass index (BMI) and female gender correlated with frailty in CHF patients [14, 15], while others reported opposite findings [8, 16]. One recent systematic review and meta-analysis evaluated the prevalence of frailty and its risk factors in CHF patients [17]. However, this research was focused on a population of older individuals, thus affecting the generalizability of its conclusions. Meanwhile, a growing body of research continues to add to the available evidence in this field, although variations in the inclusion criteria, extraction of results, and analytical methods can lead to differing outcomes for meta-analyses.

This study aimed to conduct a systematic review and meta-analysis to thoroughly assess the factors influencing frailty in CHF patients and provide an evidence-based foundation for risk prediction, treatment strategies, and improving health outcomes in clinical practice.

## 2. Methods

We performed a systematic review in accordance with the Preferred Reporting Items for Systematic Reviews and Meta-Analyses (PRISMA) checklist [18]. The standard criteria for meta-analyses of observational studies were strictly adhered to [19]. This research was registered in PROSPERO (registration number: CRD42023448771, https://www.crd.york.ac.uk/PROSPERO/view/CRD42023448771).

### 2.1 Search Strategy

We searched several databases, including the Cochrane Library, PubMed, EMBASE, CINAHL, Web of Science, CNKI, Chinese Biological Medicine (CBM), and Wan Fang up to August 25, 2024. The aim was to identify relevant reports and manually review the reference lists of retrieved articles to assemble a comprehensive collection of studies examining the factors influencing frailty in CHF patients. The search terms used were “heart failure”, “cardiac failure”, “frailty”, “frailty syndrome”, “physical frailty”, “debility”, “risk factor”, “contributing factor”, “impact factor”, “influencing factor”, “relevant factor”, “correlative factor”, “associated factor”, “predictor”, “heart disease risk factors”, and “cardiometabolic risk factors”. The PubMed database search strategy is shown in **Supplementary Material A**.

### 2.2 Selection Criteria

The inclusion criteria were as follows: (ⅰ) participants diagnosed with CHF aged 18 years or older, irrespective of pathogenesis; (ⅱ) observational studies, which could include cohort, case–control, or cross-sectional designs; (ⅲ) studies that explored the factors influencing or predicting frailty in CHF patients; (ⅳ) research that employed at least one assessment tool for frailty in the screening of CHF patients; (v) publications that provided relative risk (RR) estimates, such as hazard ratio, risk ratio, or odds ratio (OR), along with 95% confidence intervals (CIs) for at least one confounding factor; (vi) English and Chinese language publications only. The exclusion criteria were (ⅰ) unavailability of full text; (ⅱ) review, gray literature, editorials, content from non-peer-reviewed journals, letters to the editor, and study protocols; (ⅲ) studies that presented difficult data extraction or obvious errors; (ⅳ) absence of reported corresponding outcomes.

### 2.3 Data Collection and Quality Assessment

The following data were extracted from each of the included studies: first author, year of publication, country of study, survey setting, study design and sample size, participant characteristics (mean age, New York Heart Association (NYHA) class, left ventricular ejection fraction (LVEF), B-type natriuretic peptide), the frailty assessment tool or diagnostic method used, the incidence of frailty, the factors influencing frailty, and the RRs and 95% CIs. The quality of each cohort and case–control study was evaluated using the Newcastle–Ottawa scale [20], which focuses on three main areas: the selection of study groups, the comparability of these groups, and the ascertainment of either the exposure or outcome of interest in case–control or cohort studies, respectively. The cross-sectional study was assessed using the risk of bias evaluation criteria recommended by the Agency for Healthcare Research and Quality (AHRQ) [21], consisting of 11 items rated as “yes”, “no”, or “unclear”.

Two reviewers (SL, LRW) independently assessed the quality of each included study and extracted relevant data. Differing opinions were resolved through discussion or with the opinion of a third reviewer (XYX).

### 2.4 Statistical Analysis

Stata software (version 18, StataCorp LLC, College Station, TX, USA) was used to analyze all data. A chi-square test was performed to evaluate heterogeneity among studies. A result of *p* > 0.1 and *I*^2^ < 50% indicated no significant heterogeneity between studies, and in this case, the fixed-effects model was employed for analysis. However, the random-effects model was used if *p* < 0.1 and *I*^2^ ≥ 50% and in the absence of clinical heterogeneity. Statistical significance was defined as a two-sided *p*-value < 0.05. The prevalence of frailty and the odds ratios for risk factors from each study were pooled after transforming the original estimates. A sensitivity analysis was conducted on prevalence and age-related frailty in HF patients using the leave-one-out method. Additionally, a meta-regression analysis was performed to explore the potential influence of various significant factors contributing to heterogeneity. Egger’s regression asymmetry test was employed to evaluate publication bias, with a threshold of <0.05 considered to indicate the presence of bias.

## 3. Results

### 3.1 Characteristics of the Included Studies

**Fig. 1.**
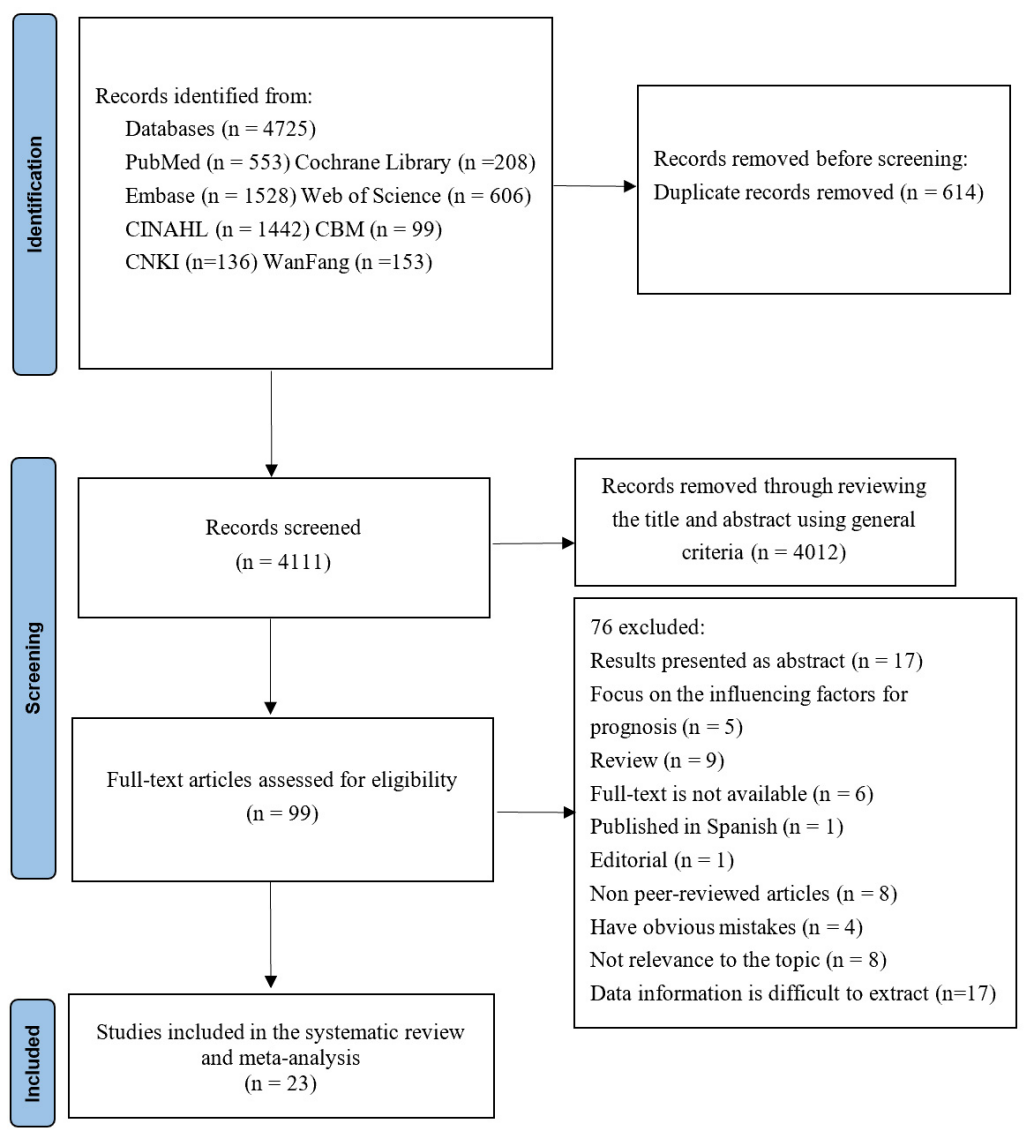
**Flow diagram for the identification of suitable studies for inclusion in this systematic review and meta-analysis**.

The database searches identified a total of 4725 records. After removing duplicates, 4111 articles remained, of which 4012 were excluded after a review of the titles and abstracts according to general screening criteria. Following the assessment of 99 full-text articles for eligibility, 76 were excluded for the following reasons: 9 were review articles, 17 presented results only in abstract form, 6 lacked full-text availability, 1 was published in Spanish, 1 was an editorial, 8 were unrelated to the topic, 8 were not peer-reviewed, 5 were focused on prognostic factors, 4 contained significant errors, and 17 contained data that was difficult to extract. The 76 excluded studies are outlined in **Supplementary Material B**. The remaining 23 articles were subjected to a systematic review and meta-analysis. Fig. 1 illustrates the detailed flowchart.

Table 1 (Ref. [14, 16, 22, 23, 24, 25, 26, 27, 28, 29, 30, 31, 32, 33, 34, 35, 36, 37, 38, 39, 40, 41, 42]) lists the details of the 23 included studies. These studies were conducted in different locations: 15 in China, two in Korea, three in Japan, two in Italy, and one in Brazil. Meanwhile, six were cohort studies, three were case–control studies, and 14 were cross-sectional studies. The included 23 studies contained 6287 patients, of whom 2622 (41.71%) were frail. The sample size of each study ranged from 76 to 949 patients, the mean age ranged from 59 to 82 years, and the most commonly utilized assessment tool was the frailty phenotype.

**Table 1.** **Characteristics of the included studies**.

First author (year)	Study design	Country	Setting	Study size	Mean ages (years)	Incidence rate (%)	LVEF (%)	NT-proBNP/BNP (pg/mL)	NYHA function class	Evaluation tool	Influence factor
Hamada (2021) [16]	Prospective cohort study	Japan	Inpatient department	949	81 (72–87)	53.70	≥50%: 244 vs. 204 (frail vs. non-frail)	BNP 278.9 (143.3–499.6)	Classes III–IV	The Kihon Checklist	A B C D E F G H I J K L
Valdiviesso (2021) [14]	Cross-sectional study	Italy	Outpatient department	136	59	15.40	Normal: 37.9 ± 13.2	Not mention	Classes I–III	Frailty phenotype	A C E M
							Prefrail: 37.0 ± 13.0				
							Frail: 41.2 ± 18.0				
Nozaki (2020) [22]	Retrospective cohort study	Japan	Inpatient department	387	74.9 ± 6.1	53.50	46.6 ± 15.8	BNP: 600 (251–1120)	Not mentioned	The frailty score	N
Komici (2020) [23]	Cross-sectional study	Italy	Inpatient department	128	69.2 ± 4.8	42.20	28.7 ± 8.5	NT-proBNP: 5922.4 ± 15,099.9	Classes III–IV	The Clinical Frailty Scale (CFS)	A B C I O E P Q H R S
Noda (2023) [24]	Retrospective cohort study	Japan	Inpatient department	922	72 (62–79)	49.90	39.0 (28.0–53.8)	BNP: 688.7 (325.0–1204.9)	Classes III–IV	The frailty score	A B C D G O R T
Son (2018) [25]	Cross-sectional Study	Korea	Outpatient department	190	70.3 ± 7.7	61.60	≤40%: 78 vs. 48 (frail vs. non-frail)	Not mentioned	Classes I II IV	The Korean version of the 5-item FRAIL	A B U V W D E X e Y Z
Ribeiro (2022) [26]	Cross-sectional study	Brazil	Outpatient department	106	68 (63.0–74.0)	28.00	34.56 ± 11.87	Not mentioned	Classes I–IV	Frailty phenotype	a Y b R
Son (2022) [27]	Cross-sectional study	Korea	Inpatient department	407	74.18 ± 7.17	28.30	58.87 ± 14.14	NT-proBNP: 342.35 ± 638.79	Classes I–IV	The Korean version of the 5-item FRAIL	A E O
Tang (2023) [28]	Prospective cohort study	China	Inpatient department	180	76.65 ± 6.64 vs. 70.20 ± 6.01 (frail vs. non-frail)	31.11	41.19 ± 8.82 vs. 47; 75 ± 8.70 (frail vs. non-frail)	Not mentioned	Classes II–IV	Frailty phenotype	A E c P H I
Quan (2017) [29]	Cross-sectional study	China	Inpatient department	371	80.5 ± 6.0	21.30	<40%: 53 vs. 192 (frail vs. non-frail)	Not mentioned	Classes II–IV	Frailty phenotype	A I K O E
Gao (2022) [30]	Case–control study	China	Inpatient department	201	74.06 ± 6.26 vs. 73.08 ± 5.80 (Frail vs. Non-frail)	23.88	57.89 ± 9.97 vs. 62.43 ± 7.56 (frail vs. non-frail)	NT-proBNP: 659.23 ± 622.51 vs. 342.83 ± 246.57 (frail vs. non-frail)	Not mentioned	The FRAIL scale	A d X K e f Z H F Q g P h
Yang (2021) [31]	Case–control study	China	Inpatient department	95	82.5 ± 7.2	32.6	63.90 ± 4.87 vs. 64.05 ± 6.04 (frail vs. non-frail)	Not mentioned	Classes II–III	Frailty phenotype	A i O F h
Yang (2022) [32]	Case–control study	China	Inpatient department	76	76.21 ± 5.82 vs. 73.18 ± 4.35 (Frail vs. Non-frail)	28.95	51.59 ± 4.07 vs. 53.76 ± 4.22 (frail vs. non-frail)	NT-proBNP: 3968.31 ± 637.24 vs. 1015.76 ± 328.85 (frail vs. non-frail)	Classes II–IV	The FRAIL scale	A E O j H F P G
Wang (2022) [33]	Cross-sectional study	China	Inpatient department	162	75.68 ± 5.67 VS 70.50 ± 5.92 (Frail vs. Non-frail)	32.72	Not mentioned	Not mentioned	Classes I–IV	Frailty phenotype	A U W k E O D
Li (2023) [34]	Cross-sectional study	China	Inpatient department	391	74 (68–80)	51.4	≥50% 139 vs. 180 (frail vs. non-frail)	Not mentioned	Classes I–IV	The FRAIL scale	l m n o p q E O D h j
Tang (2024) [35]	Cross-sectional study	China	Inpatient department	256	66.8 ± 11.2	68.75	45.5 (39, 50) vs. 54 (50, 56) (frail vs. non-frail)	4582 (3473, 6345) vs. 2430 (1363, 3580) (frail vs. non-frail)	Classes II–IV	The Tilburg Frailty Indicators scale	A o j r F Q P
Song (2023) [36]	Cross-sectional study	China	Inpatient department	223	77.68 ± 8.45	54.70	58.50 (50.00, 62.00) vs. 57.00 (44.00, 63.00) (frail vs. non-frail)	2190.00 (890.75, 6076.25) vs. 1530.00 (482.90, 5033.50) (frail vs. non-frail)	Classes III–IV	The Tilburg Frailty Indicators scale	A q O o
Tan (2024) [37]	Cross-sectional study	China	Inpatient department	198	77.56 ± 6.80 vs. 73.65 ± 7.15 (frail vs. non-frail)	32.32	53.06 ± 11.36 vs. 65.18 ± 12.76 (frail vs. non-frail)	650.38 ± 338.80 vs. 417.98 ± 261.66 (frail vs. non-frail)	Classes II–IV	The FRAIL scale	A E D O d p H I Q P s
Wang (2023) [38]	Prospective cohort study	China	Inpatient department	102	69.40 ± 5.38	53.92	44.59 ± 5.56 vs. 47.57 ± 5.59 (frail vs. non-frail)	8495.60 (4943.40, 12,588.20) vs. 4705.20 (2771.90, 7880.40) (frail vs. non-frail)	Classes II–IV	The Tilburg Frailty Indicators scale	C E O H t F Q
Zhuo (2018) [39]	Prospective cohort study	China	Inpatient department	371	73.5 ± 6.0	21.30	Not mentioned	Not mentioned	Classes II–IV	Frailty phenotype	A K I O E
She (2020) [40]	Cross-sectional study	China	Inpatient department	152	79.31 ± 7.02	25.66	Not mentioned	Not mentioned	Classes II–IV	Frailty phenotype	A O E Z
Lv (2024) [41]	Cross-sectional study	China	Inpatient department	100	75.74 ± 5.37 vs. 72.09 ± 3.89 (Frail vs. Non-frail)	54	Not mentioned	Not mentioned	Classes II–III	The Tilburg Frailty Indicators scale	A j u J
Chen (2022) [42]	Cross-sectional study	China	Inpatient department	184	76.45 ± 5.32	35.87	<40% 41.36 ± 6.20 vs. 44.28 ± 5.35 (Frail vs. Non-frail)	Not mentioned	Classes I–IV	The Elderly Frailty Assessment Scale in Chinese Version	A J E O H F I v P
					72.36 ± 6.21 (Frail vs. Non-frail)						

Notes: A, age; B, gender; C, body mass index (BMI); D, prior HF hospitalization; E, NYHA function class; F, albumin; G, B-type natriuretic peptide BNP; H, hemoglobin; I, renal insufficiency; J, living alone; K, cerebrovascular accident (CVA); L, atrial fibrillation; M, middle arm muscle circumference; N, rising time; O, comorbidity; P, LVEF; Q, NT-proBNP N-terminal pro-B-type natriuretic peptide; R, C-reactive protein (CRP); S, galectin-3 (Gal-3); T, hepato-renal dysfunction; U, education; V, unemployed; W, unmarried; X, hypertension; Y, no diuretics; Z, depressed; a, no β-blocker; b, functional capacity; c, aortic dimension; d, duration of hospitalization; e, diabetes; f, chronic obstructive pulmonary disease (COPD); g, high-density lipoprotein cholesterol (HDL-C); h, left atrial diameter; i, polypharmacy; j, nutrition risk; k, income/month; l, drinking; m, grip strength; n, Barthel Index; o, instrumental activities of daily living; p, Pittsburgh Sleep Quality Index; q, Morse Fall scale; r, the score of The Chinese version of the Tampa Scale for Kinesiophobia Heart (TSK-SV Heart-C); s, self-care ability; t, increased red cell distribution width (RDW); u, mini-mental state examination (MMSE); V, low-density lipoprotein cholesterol (LDL-C); LVEF, left ventricular ejection fraction; NT-proBNP/BNP, N-terminal pro-B-type natriuretic peptide; BNP, B-type natriuretic peptide; NYHA, New York Heart Association.

### 3.2 Quality Assessment

The quality of each included study was assessed using the tools described in the Methods. For most included studies, the overall risk of bias was considered acceptable (**Supplementary Material C**).

### 3.3 Prevalence of Frailty in CHF Patients

**Fig. 2.**
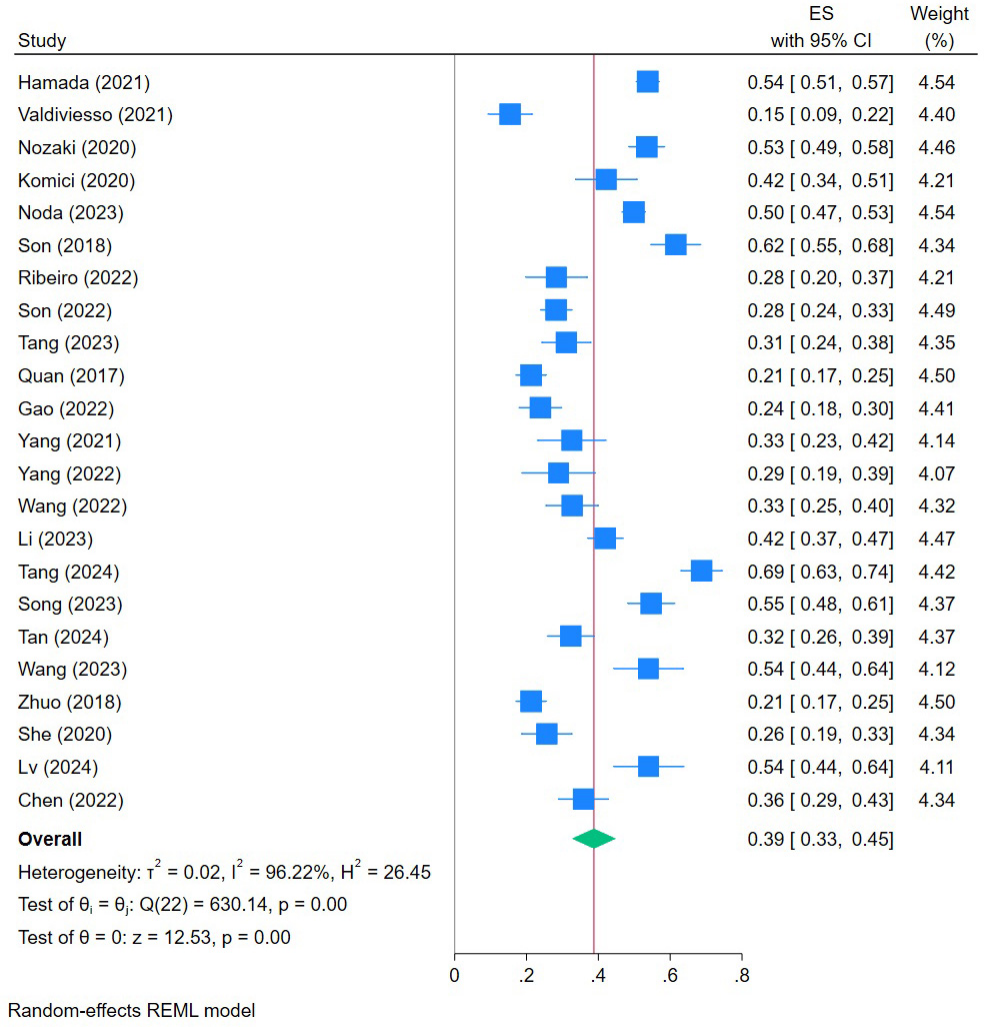
**Forest plot of the prevalence of frailty in CHF patients**. ES, effect size; REML, restricted maximum likelihood.

The prevalence of frailty in CHF patients in the 23 studies suitable for meta-analysis ranged from 15.40% to 68.75%. From the random-effects model-based meta-analysis conducted on all data points, the overall prevalence of frailty was estimated to be 39% (95% CI: 0.33–0.45, *I*^2^ = 96.22%, *p* < 0.0001) (Fig. 2). Subgroup analysis revealed the prevalence of frailty was significantly higher in developed countries (44%) than in developing countries (37%). Comparing different assessment tools, the prevalence of frailty was 58% using the Tilburg Frailty Indicator scale and 25% using the frailty phenotype. With regard to the study design, the prevalence of frailty was highest in the retrospective cohort studies (51%) and lowest in the case–control studies (27%) (Table 2). **Supplementary Material D** shows detailed forest maps for each subgroup. Next, we conducted a meta-regression analysis of prevalence based on covariates such as age, study region, frailty assessment tools, study design, and publication year. This revealed that the assessment tools were the source of heterogeneity in the study (Table 3).

**Table 2.** **Subgroup analyses were performed for the pooled prevalence of frailty by age, study region, assessment tools, study design, and publication year**.

Subgroup		Number of studies	Sample size	Frailty	Pooled prevalence (95% CI)	Heterogeneity	
						*I*^2^ (%)	*p*-value
Age (y)							
	<60	1	136	21	15% (0.09–0.22)	-	-
	61–70	4	592	315	48% (0.31–0.66)	94.60	0.00
	71–80	15	4144	1666	38% (0.32–0.45)	95.11	0.00
	81–90	3	1415	620	36% (0.17–0.55)	97.91	0.00
Study region							
	Developed countries	7	3119	1484	44% (0.31–0.56)	97.86	0.00
	Developing countries	16	3168	1138	37% (0.30–0.44)	94.62	0.00
Assessment tools							
	Frailty phenotype	8	1573	388	25% (0.21–0.30)	72.11	0.00
	The FRAIL scale	4	866	298	32% (0.23–0.41)	86.71	0.00
	The Tilburg Frailty Indicators scale	4	681	407	58% (0.50–0.67)	79.44	0.00
	The Clinical Frailty scale	1	128	54	42% (0.34–0.51)	-	-
	Others	6	1730	3039	47% (0.38–0.56)	95.95	0.00
Study design							
	Prospective cohort study	4	1602	700	40% (0.21–0.59)	98.16	0.00
	Retrospective cohort study	2	1309	667	51% (0.48–0.55)	29.35	0.23
	Case–control study	3	372	101	27% (0.22–0.33)	22.54	0.27
	Cross-sectional study	14	3004	1154	39% (0.30–0.47)	96.28	0.00
Publication year							
	≤2020 year	6	1599	575	37% (0.23–0.52)	97.50	0.00
	>2020 year	17	4688	2047	39% (0.32–0.46)	95.81	0.00

**Table 3.** **Meta-regression analysis results for the prevalence of frailty in patients with CHF**.

Covariate	β	SE	95% CI	*p*-value
Publication year	–0.026	0.055	–0.142, 0.091	0.649
Age	–0.021	0.035	–0.095, 0.053	0.557
Study design	–0.010	0.027	–0.066, 0.046	0.711
Study region	0.093	0.063	–0.039, 0.225	0.154
Assessment tools	0.079	0.017	0.044, 0.114	0.000

Note: CHF, chronic heart failure.

### 3.4 Risk Factors for Frailty in CHF Patients

A total of 48 relevant factors were identified for frailty across the studies included in this analysis. From these, 24 factors that influenced frailty in CHF patients were selected from 22 studies for the meta-analysis. Older age, cerebrovascular accidents, longer duration of hospitalization, larger left atrial diameter, higher number of comorbidities, poor NYHA functional class, and poor sleep quality were shown to be positively associated with frailty in CHF. Conversely, higher albumin, hemoglobin, and LVEF levels were negatively associated with frailty (Table 4). We also conducted a meta-regression analysis of age based on covariates such as publication year, study design, study region, and frailty assessment tool. As shown in Table 5, no sources of heterogeneity were found. Detailed forest maps for each risk factor are shown in **Supplementary Material E**.

**Table 4.** **Pooled relevant factors for frailty in CHF patients**.

Influence factor	Heterogeneity			Model	OR	95% CI	*p*-value
	*I*^2^ (%)	*Q*	*p*-value				
Age	77	73.80	<0.01	Random	1.12	1.08, 1.17	<0.01
Albumin	30	5.69	0.22	Fixed	0.81	0.74, 0.89	0.00
Cerebrovascular accidents	0	0.84	0.84	Fixed	2.08	1.51, 2.85	0.00
Hemoglobin	78	22.26	0.00	Random	0.91	0.84, 0.98	0.02
Duration of hospitalization	0	0.14	0.71	Fixed	1.14	1.06, 1.23	<0.01
Left atrial diameter	22	2.55	0.28	Fixed	1.06	1.02, 1.10	<0.01
LVEF	61	10.29	0.04	Random	0.91	0.87, 0.96	0.00
Number of comorbidities	98	336.11	0.00	Random	2.16	1.08, 4.33	0.03
NYHA functional class	24	17.15	0.19	Fixed	2.81	2.35, 3.36	<0.01
Sleep quality	10	1.11	0.29	Fixed	1.78	1.13, 2.80	0.01

Notes: OR, odds ratio.

**Table 5.** **Results of meta-regression analysis for age**.

Covariate	β	SE	95% CI	*p*-value
Publication year	–0.030	0.125	–0.301, 0.241	0.817
Study design	–0.019	0.052	–0.132, 0.094	0.724
Study region	–0.062	0.152	–0.390, 0.266	0.690
Assessment tools	–0.068	0.044	–0.163, 0.026	0.142

### 3.5 Sensitivity Analysis and Publication Bias

After removing each individual trial, the sensitivity analysis revealed that the pooled prevalence of frailty and the effect of age on frailty in patients with CHF did not significantly change, indicating the stability of the meta-analysis results (**Supplementary Material F**). The analysis revealed a low probability of publication bias for the prevalence of frailty (Egger’s test, *p* = 0.539), as shown by the funnel plot. However, evidence of publication bias was observed for the effect of age on frailty in patients with CHF (Egger’s test, *p* < 0.001). This result remained unchanged after evaluation using the trimming method (*p* < 0.05), indicating the relative stability of the combined results (**Supplementary Material G**).

## 4. Discussion

To our knowledge, this is the first comprehensive review and meta-analysis to systematically assess the prevalence of frailty in CHF patients and the factors influencing frailty beyond just an older population. Our findings revealed the prevalence of frailty among CHF patients was 39%. Furthermore, the factors found to be positively associated with frailty in CHF were older age, cerebrovascular accidents, longer hospital stays, large left atrial diameter, high number of comorbidities, poor NYHA functional class, and poor sleep quality. Conversely, higher albumin, hemoglobin, and LVEF levels were negatively associated with frailty.

The prevalence of frailty among CHF patients found in this study (39%) was consistent with the findings reported by Li *et al*. [17], thus confirming a significant characteristic across all age groups. Denfeld *et al*. [10] and Davis *et al*. [43] reported that the prevalence of frailty differed between multidimensional and physical frailty measures, which also agrees with our results. In addition, meta-regression analysis conducted in the present study revealed the assessment tools were a source of heterogeneity. The subgroup analysis in our study showed that the prevalence of frailty was significantly higher in developed countries than in developing countries, which contrasts with the results from previous studies [44, 45]. The developed countries of Italy and Japan are classified as super-aged societies, while South Korea is considered an aged society. These countries have a high prevalence of HF and frailty, and it is well-known that the likelihood of developing frailty increases with age. Hence, the interaction between super-aged populations and HF contributes to the high prevalence of frailty in these developed countries. However, the prevalence of frailty in HF patients from different age groups does not show a clear trend. Instead, our analysis suggests this variability is influenced by a combination of geographical factors and the assessment tools employed, possibly explaining the discordant conclusions. Among the included studies, most of the CHF patients aged >70 years were from China. The large population base in China creates significant challenges for many patients when trying to access medical care. Consequently, the clinical focus is more on HF rather than on frailty, and the screening for frailty may be insufficient and incomplete, leading to missed diagnoses.

Our finding that age was an influential factor associated with frailty in CHF patients was similar to that by He *et al*. [46] and McAlister [47]. Meanwhile, a systematic review indicated that older patients with HF were 6–7-fold more likely to be frail compared to younger patients [48]. Thus, the older the CHF patient, the higher the risk of frailty. Additionally, as people age, they generally reduce the amount of daily living activities. Hence, with the decreased activity tolerance of CHF patients and poor immune function, these changes eventually lead to frailty [49]. The aging process is associated with DNA damage, reduced autophagy, and increased oxidative stress resulting from mitochondrial dysfunction, which may be considered an accelerated form of frailty in CHF patients [50]. Alternatively, the heart undergoes age-related changes as individuals age, including structural alterations such as ventricular hypertrophy and reduced diastolic and systolic function. Such changes negatively impact the heart’s pumping ability, leading to increased energy expenditure and a reduced capacity to respond to stress in patients with CHF, ultimately contributing to the development of frailty. In addition, the aging heart is associated with chronic low-grade inflammation, which exacerbates cardiac dysfunction and facilitates the onset of frailty [51]. Simultaneously, frailty was shown to accelerate cardiac aging, resulting in fibrosis and slowed conduction in the sinoatrial node and atria, which further contributes to the onset and worsening of HF [52].

Our results showed that a poor NYHA classification was significantly linked to frailty in patients with CHF. Individuals with compromised cardiac function tend to limit their physical activity, which can result in skeletal muscle atrophy and an increased risk of frailty [10]. Evidence suggests that engaging in physical activities such as resistance training can help to reverse or slow muscle wasting [53]. Therefore, implementing rehabilitation exercises during periods of stable patient conditions may enhance functional capacity and potentially prevent or mitigate the progression of frailty.

The results of this systematic review and meta-analysis suggest that effective nutritional management and support could play a significant role in lowering the risk of frailty among CHF patients. Our analysis revealed that higher levels of albumin and hemoglobin were protective factors against frailty in HF patients. Conversely, those at risk of malnutrition were found to be more susceptible to frailty compared to their counterparts. A cross-sectional study by Chaves *et al*. [54] found that mildly low and low–normal hemoglobin levels were independently associated with an increased risk of frailty in community-dwelling older women. Additionally, cardiovascular diseases such as CHF decrease the ability to mount compensatory reactions to anemia, leading to increased susceptibility to frailty. Malnutrition contributes to a reduced rate of protein synthesis and the loss of muscle mass, thereby increasing the risk and progression of sarcopenia, a key factor in the development of frailty [55].

The present study also found that multimorbidity contributes to frailty in patients with CHF, which agrees with the results reported by Vetrano *et al*. [56]. The presence of multiple chronic conditions significantly affects the progression of frailty, thus complicating the management of these diseases in frail individuals. Tazzeo *et al*. [57] reported that among the various patterns of multimorbidity, cardiovascular and neuropsychiatric disorders were most strongly linked to physical frailty, regardless of whether the analysis was cross-sectional or longitudinal. Additionally, our results demonstrated that cerebrovascular accidents significantly increased the risk of frailty in CHF patients. This highlights the importance of implementing an early warning system for HF patients with cerebrovascular conditions and adopting targeted interventions to prevent frailty. CHF patients with multiple comorbidities often rely on polypharmacy. Gnjidic and Hilmer [58] proposed several plausible mechanisms through which drugs could affect the development of frailty, including weight loss, balance disorders, and functional deterioration. For example, some medications can induce low blood pressure in patients; however, while this may initially reduce the heart’s workload, prolonged low blood pressure can result in insufficient blood supply to the heart and other vital organs. This can cause heart compensation mechanisms to fail, leading to frailty. Hypotension can increase the risk of falls; meanwhile, after a fall, the patient may require hospitalization or bed rest, which can lead to the development of frailty. For CHF patients, using diuretics poses the risk of electrolyte imbalance (sodium, potassium, magnesium, etc.), which plays a critical role in heart function and muscle contraction [59]. This imbalance can lead to muscle weakness and impair the heart’s pumping ability, further exacerbating feelings of fatigue and weakness.

In our systematic review, Nozaki *et al*. [22] reported that the rising time from bed, when cardiac rehabilitation was started within 2 days after admission, was independently associated with higher prevalence of frailty in HF patients [22]. The longer rising time from bed, the higher the risk of frailty. These results suggest that evaluating the increase in time could be useful in detecting reduced physical performance.

Our study did not identify a significant difference in frailty between genders. Conversely, most prior studies have reported that frailty is more prevalent in females than males [46, 60]. However, our selection criteria meant that the number of articles included for analysis was limited. Moreover, the study designs (cross-sectional and retrospective cohort) made it difficult to observe gender differences in frailty over long periods.

Previous studies have reported a link between increased inflammation and frailty, independent of significant clinical comorbidities [61, 62]. However, in the present analysis, C-reactive phase protein was not significantly associated with frailty in HF patients. Variations in the detection techniques and analytical methods used in the three studies [22, 23, 24] may have contributed to the discordant conclusions.

Fried *et al*. [6] reported that psychiatric conditions such as depression and anxiety can adversely impact physical frailty in patients with HF. After adjusting for other confounding variables, Son and Seo [25] identified depression as an independent determinant of frailty in HF patients. However, no significant correlation was found between depression and frailty in the current study. This may be explained by the relatively small sample size and heterogeneity between different studies. Further research on the relationship between frailty and depression in various contexts and populations could help to clarify the role of depression in frailty among HF patients and identify potential targets for intervention.

## 5. Limitations and Strength

This review examined factors that affect frailty in CHF patients. The relevant literature included various study designs, geographic locations, and ethnic groups. However, our analysis was limited to published literature in the English or Chinese language, which may have resulted in an incomplete collection of studies. Thus, future research should explore additional languages and the grey literature. Furthermore, the variability in study design, sample size, and assessment tools requires the performance of more robust, well-structured, and standardized studies. Additionally, the factors investigated for their influence on frailty were dispersed across different studies, thus preventing some of them from being evaluated by the meta-analysis.

## 6. Conclusions

The prevalence of frailty in patients with CHF is relatively high and varies depending on the assessment tool applied. Therefore, establishing specific frailty assessment tools for HF patients and providing targeted interventions based on important risk factors are essential for improving outcomes and reducing the burden of frailty.

## Availability of Data and Materials

All data analyzed in this study are available from the corresponding author upon a reasonable request.

## Supplementary Material

Supplementary material associated with this article can be found, in the online version, at https://doi.org/10.31083/RCM26854.
