# Supplementary material for: Prevalence and Associated Factors of Frailty in Patients with Chronic Heart Failure: A Systematic Review and Meta-Analysis
Source: Rev Cardiovasc Med. 2025 Mar 24;26(3):26854. doi: 10.31083/RCM26854 (PMC11951493; doi:10.31083/RCM26854)
Supplement: Supplementary file 1 [file 2153-8174-26-3-26854-s1.zip › Additional file 2 PROSPERO systematic reviews register.pdf]

National Institute for Health Research

UNIVERSITY of York
Centre for Reviews and Dissemination

Systematic review

A list of fields that can be edited in an update can be found here

1. * Review title.

Give the title of the review in English

Prevalence and risk factors of frailty syndrome in elderly patients with chronic heart failure: a systematic review and meta-analysis

2. Original language title.

For reviews in languages other than English, give the title in the original language. This will be displayed with the English language title.

3. * Anticipated or actual start date.

Give the date the systematic review started or is expected to start.

10/08/2023

4. * Anticipated completion date.

Give the date by which the review is expected to be completed.

10/11/2023

5. * Stage of review at time of this submission.

This field uses answers to initial screening questions. It cannot be edited until after registration.

Tick the boxes to show which review tasks have been started and which have been completed.

Update this field each time any amendments are made to a published record.

The review has not yet started: Yes

Review stage	Started	Completed
Preliminary searches	No	No
Piloting of the study selection process	No	No
Formal screening of search results against eligibility criteria	No	No
Data extraction	No	No
Risk of bias (quality) assessment	No	No
Data analysis	No	No

Provide any other relevant information about the stage of the review here.

6. * Named contact.

The named contact is the guarantor for the accuracy of the information in the register record. This may be any member of the review team.

Si Liu

Email salutation (e.g. "Dr Smith" or "Joanne") for correspondence:

Mrs Liu

7. * Named contact email.

Give the electronic email address of the named contact.

1028970010@qq.com

8. Named contact address

Give the full institutional/organisational postal address for the named contact.

School of Nursing, Nan Chang University, Nan Chang, Jiang Xi, People’s Republic of China

9. Named contact phone number.

Give the telephone number for the named contact, including international dialling code.

+86-18870039387

10. * Organisational affiliation of the review.

Full title of the organisational affiliations for this review and website address if available. This field may be

completed as 'None' if the review is not affiliated to any organisation.

Nan Chang University

Organisation web address:

11. * Review team members and their organisational affiliations.

Give the personal details and the organisational affiliations of each member of the review team. Affiliation refers to groups or organisations to which review team members belong. **NOTE: email and country now MUST be entered for each person, unless you are amending a published record.**

Si Liu. School of Nursing, Nan Chang University
Mengdie Liu. School of Nursing, Nan Chang University
Hua Chen. School of Nursing, Nan Chang University
Ying Wang. School of Nursing, Nan Chang University
Ying Yang. School of Nursing, Nan Chang University
Meijun Zhang. School of Nursing, Nan Chang University
Qin Xiang. School of Nursing, Nan Chang University
Xiaoyun Xiong. Nursing Department, The Second Affilated Hospital of Nanchang University

12. * Funding sources/sponsors.

Details of the individuals, organizations, groups, companies or other legal entities who have funded or sponsored the review.

No

Grant number(s)

State the funder, grant or award number and the date of award

13. * Conflicts of interest.

List actual or perceived conflicts of interest (financial or academic).

None

14. Collaborators.

Give the name and affiliation of any individuals or organisations who are working on the review but who are not listed as review team members. **NOTE: email and country must be completed for each person, unless you are amending a published record.**

15. * Review question.

State the review question(s) clearly and precisely. It may be appropriate to break very broad questions down into a series of related more specific questions. Questions may be framed or refined using PI(E)COS or similar where relevant.

What are the prevalence and risk factors of frailty syndrome in elderly patients with chronic heart failure?

16. * Searches.

State the sources that will be searched (e.g. Medline). Give the search dates, and any restrictions (e.g. language or publication date). Do NOT enter the full search strategy (it may be provided as a link or attachment below.)

We will search EMBASE, the Cochrane Library, PubMed, Web of Science, the Chinese Biological Medicine Database(CBM), the Wanfang Database and the China National Knowledge Infrastructure (CNKI) database from inception to August 26, 2023.

17. URL to search strategy.

Upload a file with your search strategy, or an example of a search strategy for a specific database, (including the keywords) in pdf or word format. In doing so you are consenting to the file being made publicly accessible. Or provide a URL or link to the strategy. Do NOT provide links to your search **results**.

Alternatively, upload your search strategy to CRD in pdf format. Please note that by doing so you are consenting to the file being made publicly accessible.

Do not make this file publicly available until the review is complete

18. * Condition or domain being studied.

Give a short description of the disease, condition or healthcare domain being studied in your systematic review.

Similar to most patients with chronic diseases, patients with chronic heart failure typically have multiple comorbidities, complex medication regimens and limited self-management skills. Therefore, chronic heart failure is characterized by a high risk of morbidity, a high risk of deterioration, a high risk of mortality and the highest risk of early readmission. Frailty is a complexage-related clinical condition characterised by a decline in physiological capacity across several organ systems, with a resultant increased susceptibility to stressors. Although frailty has been associated with increased risks for hospitalization and mortality in chronic heart failure, the risk factor of frailty in elderly patients with chronic heart failure remains uncertain, and an updated systematic review and meta-analysis to assess the prevalence of frailty in heart failure is not perform. Therefore, the purpose of this review is to summarize risk factors that cause frailty in elderly patients with

chronic heart failure and to update systematic review and meta-analysis to assess the prevalence

of frailty in elderly patients with chronic heart failure.

19. * Participants/population.

Specify the participants or populations being studied in the review. The preferred format includes details of both inclusion and exclusion criteria.

Patients over 60 years of age with chronic heart failure.

20. * Intervention(s), exposure(s).

Give full and clear descriptions or definitions of the interventions or the exposures to be reviewed. The preferred format includes details of both inclusion and exclusion criteria.

frailty syndrome

21. * Comparator(s)/control.

Where relevant, give details of the alternatives against which the intervention/exposure will be compared (e.g. another intervention or a non-exposed control group). The preferred format includes details of both inclusion and exclusion criteria.

non-frail patients

22. * Types of study to be included.

Give details of the study designs (e.g. RCT) that are eligible for inclusion in the review. The preferred format includes both inclusion and exclusion criteria. If there are no restrictions on the types of study, this should be stated.

We will include case-control studies and cohort studies.

23. Context.

Give summary details of the setting or other relevant characteristics, which help define the inclusion or exclusion criteria.

24. * Main outcome(s).

Give the pre-specified main (most important) outcomes of the review, including details of how the outcome is defined and measured and when these measurement are made, if these are part of the review inclusion criteria.

Prevalence and risk factors, such as gender, NYHA, HFpEF, education level, malnutrition and so on.

Measures of effect

Please specify the effect measure(s) for you main outcome(s) e.g. relative risks, odds ratios, risk difference, and/or 'number needed to treat.

25. * Additional outcome(s).

List the pre-specified additional outcomes of the review, with a similar level of detail to that required for main outcomes. Where there are no additional outcomes please state 'None' or 'Not applicable' as appropriate to the review

None.

Measures of effect

Please specify the effect measure(s) for you additional outcome(s) e.g. relative risks, odds ratios, risk difference, and/or 'number needed to treat.

26. * Data extraction (selection and coding).

Describe how studies will be selected for inclusion. State what data will be extracted or obtained. State how

this will be done and recorded.

Two review authors will independently screen and extract data like study design, study size, LVEF, NYHA, etal. Any discrepancies will be identified and resolved through discussion and by referring back to the original article. When necessary, information will be sought from the authors of the primary studies. We will consult a third author to resolve any disagreement.

27. * Risk of bias (quality) assessment.

State which characteristics of the studies will be assessed and/or any formal risk of bias/quality assessment tools that will be used.

Two authors will assesse the risk of bias for the studies included using the Newcastle–Ottawa Scale (NOS).The terms to evaluate case-control studies and cohort studies in this scale are different. There are three terms for case-control studies, including"Selection", "Comparability" and "Exposure". The terms for cohort studies are "Selection","Comparability" and "Outcome". Differing opinions were resolved through discussion or a third reviewer's judgment.

28. * Strategy for data synthesis.

Describe the methods you plan to use to synthesise data. This **must not be generic text** but should be **specific to your review** and describe how the proposed approach will be applied to your data. If meta-analysis is planned, describe the models to be used, methods to explore statistical heterogeneity, and software package to be used.

Odds ratio (OR) and 95%CI were extracted. I^2test (Q test) was used to determine the heterogeneity of the results. If P0.1 and $I^2$50%, indicating that the statistical heterogeneity between studies was acceptable, and the fixed effect model was used for meta-analysis. if P0.1, I^2?50% and in the absence of clinical heterogeneity, we chose the random-effects model. When the number of included papers exceeds 10, the funnel plot should be used for publication bias analysis.

29. * Analysis of subgroups or subsets.

State any planned investigation of ‘subgroups’. Be clear and specific about which type of study or participant will be included in each group or covariate investigated. State the planned analytic approach.

If available, we will plan to conduct subgroup analyses.

30. * Type and method of review.

Select the type of review, review method and health area from the lists below.

Type of review

Cost effectiveness

No

Diagnostic

No

Epidemiologic

Yes

Individual patient data (IPD) meta-analysis

No

Intervention

No

Living systematic review

No

Meta-analysis

Yes

Methodology

No

Narrative synthesis

No

Network meta-analysis

No

Pre-clinical

No

Prevention

No

Prognostic

No

Prospective meta-analysis (PMA)

No

Review of reviews

No

Service delivery

No

Synthesis of qualitative studies

No

Systematic review

Yes

Other

No

Health area of the review

Alcohol/substance misuse/abuse

No

Blood and immune system

No

Cancer

No

Cardiovascular

Yes

Care of the elderly

No

Child health

No

Complementary therapies

No

COVID-19

No

Crime and justice

No

Dental

No

Digestive system

No

Ear, nose and throat

No

Education

No

Endocrine and metabolic disorders

No

Eye disorders

No

General interest

No

Genetics

No

Health inequalities/health equity

No

Infections and infestations

No

International development
No

Mental health and behavioural conditions
No

Musculoskeletal
No

Neurological
No

Nursing
Yes

Obstetrics and gynaecology
No

Oral health
No

Palliative care
No

Perioperative care
No

Physiotherapy
No

Pregnancy and childbirth
No

Public health (including social determinants of health)
No

Rehabilitation
No

Respiratory disorders
No

Service delivery
No

Skin disorders
No

Social care
No

Surgery

No

Tropical Medicine

No

Urological

No

Wounds, injuries and accidents

No

Violence and abuse

No

31. Language.

Select each language individually to add it to the list below, use the bin icon to remove any added in error.

English

There is an English language summary.

32. * Country.

Select the country in which the review is being carried out. For multi-national collaborations select all the countries involved.

China

33. Other registration details.

Name any other organisation where the systematic review title or protocol is registered (e.g. Campbell, or The Joanna Briggs Institute) together with any unique identification number assigned by them. If extracted data will be stored and made available through a repository such as the Systematic Review Data Repository (SRDR), details and a link should be included here. If none, leave blank.

34. Reference and/or URL for published protocol.

If the protocol for this review is published provide details (authors, title and journal details, preferably in Vancouver format)

Add web link to the published protocol.

Or, upload your published protocol here in pdf format. Note that the upload will be publicly accessible.

No I do not make this file publicly available until the review is complete

Please note that the information required in the PROSPERO registration form must be completed in full even if access to a protocol is given.

35. Dissemination plans.

Do you intend to publish the review on completion?

Yes

Give brief details of plans for communicating review findings.?

36. Keywords.

Give words or phrases that best describe the review. Separate keywords with a semicolon or new line. Keywords help PROSPERO users find your review (keywords do not appear in the public record but are included in searches). Be as specific and precise as possible. Avoid acronyms and abbreviations unless these are in wide use.

37. Details of any existing review of the same topic by the same authors.

If you are registering an update of an existing review give details of the earlier versions and include a full bibliographic reference, if available.

38. * Current review status.

Update review status when the review is completed and when it is published.New registrations must be ongoing so this field is not editable for initial submission.

Please provide anticipated publication date

Review_Ongoing

39. Any additional information.

Provide any other information relevant to the registration of this review.

40. Details of final report/publication(s) or preprints if available.

Leave empty until publication details are available OR you have a link to a preprint (NOTE: this field is not editable for initial submission). List authors, title and journal details preferably in Vancouver format.

Give the link to the published review or preprint.
