# Supplementary material for: Prevalence and Associated Factors of Frailty in Patients with Chronic Heart Failure: A Systematic Review and Meta-Analysis
Source: Rev Cardiovasc Med. 2025 Mar 24;26(3):26854. doi: 10.31083/RCM26854 (PMC11951493; doi:10.31083/RCM26854)

Supplementary Material A: Search Strategy Example

**PubMed search：**

("Heart Failure"[MeSH Terms] OR "heart failure, diastolic"[MeSH Terms] OR "heart failure, systolic"[MeSH Terms] OR ("Heart Failure"[Title/Abstract] OR "cardiac failure"[Title/Abstract])) AND ("Frailty"[MeSH Terms] OR ("Frailty"[Title/Abstract] OR "debility"[Title/Abstract] OR "frailty syndrome"[Title/Abstract] OR "physical frailty"[Title/Abstract])) AND ("Risk Factors"[MeSH Terms] OR "Cardiometabolic Risk Factors"[MeSH Terms] OR "Heart Disease Risk Factors"[MeSH Terms] OR ("risk factor*"[Title/Abstract] OR "contributing factor*"[Title/Abstract] OR "impact factor*"[Title/Abstract] OR "influencing factor*"[Title/Abstract] OR "relevant factor*"[Title/Abstract] OR "relative factor*"[Title/Abstract] OR "correlative factor*"[Title/Abstract] OR "associated factor*"[Title/Abstract] OR "predictor*"[Title/Abstract]))

Supplementary Material B: Reasons for final exclusion 76 studies

**Review(n=9)**

1. Untangling the Sex-Frailty Paradox in Heart Failure
2. The Role of Depression and Anxiety in Frail Patients with Heart Failure
3. Frailty in advanced heart failure: a systematic review
4. Prognosis of Frailty in Patients with Heart Failure...Y. Zhang M. Yuan M. Gong Frailty and clinical outcomes in heart failure: A systematic review and meta-analysis Am Med Dir Assoc 19 2018 1003 1008.e1
5. The challenge of frailty and sarcopenia in heart failure with preserved ejection fraction
6. Physical frailty in older patients with acute heart failure: From risk marker to modifiable treatment target
7. Multidimensional frailty evaluation in elderly outpatients with chronic heart failure: A prospective study
8. Heart Failure and Problems with Frailty Syndrome: Why it is Time to Care About Frailty Syndrome in Heart Failure
9. Coexisting Frailty with Heart Failure

**Editorial(n=1)**

1.Body mass loss is a surrogate marker of frailty in heart failure

**Results presented as abstract(n=17)**

1. Cardiopulmonary exercise testing for assessing frailty status in stable elderly patients with heart failure
2. Mid and Later Life Reproductive Factors Are Not Associated with Physical Frailty Among Women with Heart Failure
3. Women are More Likely to be Physically Frail Compared with Men in Heart Failure
4. Frailty in heart failure: Prevalence and factors associated in elderly patients hospitalized in Santiago, Chile
5. Frailty phenotype is associated with decreased survival as predicted by the Seattle heart failure model and adverse outcomes in heart failure patients referred for advanced therapies
6. Determinants of frailty in individuals with heart failure
7. Prevalence and clinical impact of social frailty in elderly patients hospitalized for acute decompensated heart failure: A multicenter prospective cohort study
8. Assessing the relationship between frailty syndrome and self-care in chronic heart failure
9. The association between frailty and physical performance in elderly patients with heart failure
10. HEART FAILURE SELF-CARE IN VULNERABLE OLDER ADULTS: A CROSS-SECTIONAL ANALYSIS OF THE FRAILTY-HF STUDY
11. Frailty quick test, effective for risk stratification of inadequate treatment of heart failure
12. Loss of social role awareness, a subdomain of social frailty, is an independent predictor of future adverse events in hospitalized older patients with heart failure
13. Low blood pressure in heart failure patients with underweight is the predictive factor of mortality and risk factor of frailty
14. Assessment of frailty diagnosed by simple index in hospitalized acute heart failure patients
15. The influence of frailty syndrome using the tilburg frailty indicator (TFI) on the assessment of illness acceptance in elderly patients with chronic heart failure
16. FRAILTY PREVALENCE AND RISK OF SARCOPENIA IN OLDER HEART FAILURE (HF) INPATIENTS
17. Comparison of Frailty Phenotype and Six Minute Walk as Predictors of Survival and Performance in Heart Failure

**Full-text is not available(n=6)**

1. Heart failure, older people and frailty
2. Frailty in patients admitted to hospital with acute decompensated heart failure
3. Physical frailty and its impact on long-term outcomes in older patients with acute heart failure after discharge from an emergency department
4. Mining big data to estimate the frailty index in patients with congestive heart failure: Clinical expert vs machine learning
5. Clinical Characteristics and Social Frailty of Super-Elderly Patients With Heart Failure
6. Frailty in patients with heart failure

**Focus on the influencing factors for prognosis(n=5)**

1. Frailty and prognosis of older patients with chronic heart failure
2. Prevalence and prognostic impact of frailty and its components in non-dependent elderly patients with heart failure
3. Sex differences in the prevalence and prognostic impact of physical frailty and sarcopenia among older patients with heart failure
4. Prevalence and prognostic impact of the coexistence of multiple frailty domains in elderly patients with heart failure the FRAGILE-HF cohort study
5. Frailty is associated with 90-day unplanned readmissions and death in patients with heart failure: A longitudinal study in China

**Published in Spanish (n=1)**

1.Frailty in patients admitted to hospital with acute decompensated heart failure

**Non peer-reviewed articles(n=8)**

1. Effects of Different Sleep Quality Dimensions on Frailty in Elderly Patients with Chronic Heart Failure
2. The influencing factors analysis of frailty in hospitalized elderly patients with chronic heart failure
3. Study on the Correlation between Soluble Suppression of Tumorigenicity 2 and Frailty in the Patients with Chronic Heart Failure
4. Analysis of Risk Factors in Elderly Patients with Heart Failure and Frailty
5. Influencing factors and intervention of senile heart failure patients frailty
6. Study on the correlation between heart failure (HF) patients and debilitating and serum cholinesterase
7. The association between dietary patterns and frailty in patients with chronic heart failure
8. Study on the Relationship between eGFR and Frailty in the Patients with Chronic Heart Failure

**Have mistakes (n=4)**

1. Analysis of status of frailty in elderly hospitalized patients with cardiac insufficiency and the correlation
2. The influencing factors analysis of frailty in patients with chronic heart failure in stable stage
3. Analysis of risk factors for frailty in elderly patients with heart failure
4. Factors influencing frailty in patients with stable chronic heart failure

**Not relevance to the topic(n=8)**

1. Frailty Syndrome in Heart Failure Patients who are Receiving Cardiac Resynchronization
2. Frailty implications for exercise participation and outcomes in patients with heart failure
3. Identification of Frailty in Chronic Heart Failure
4. Frailty phenotype in heart failure: A condition that transcends age
5. Multidimensional frailty evaluation in elderly outpatients with chronic heart failure: A prospective study
6. The 6-minute walk is associated with frailty and predicts mortality in older adults with heart failure
7. The utility of the 6-minute walk test as a measure of frailty in older adults with heart failure
8. Clinical Characteristics and Social Frailty of Super-Elderly Patients With Heart Failure

[**Data information**](javascript:;) **is difficult to extract(n=17)**

1. Association of Diagnostic Coding-Based Frailty and Outcomes in Patients With Heart Failure: A Report From the Veterans Affairs Health System
2. Aspartate aminotransferase to alanine aminotransferase ratio is associated with frailty and mortality in older patients with heart failure
3. Frailty syndrome and self-care ability in elderly patients with heart failure
4. Disentangling Heart Failure and Physical Frailty: Prospective Study of Patients Undergoing Percutaneous Mitral Valve Repair
5. Frailty and predictive factors in Chinese hospitalized patients with heart failure: a structural equation model analysis
6. Candidate biomarkers of physical frailty in heart failure: an exploratory cross-sectional study
7. Frailty Affects Self-Care Behavior in Congestive Heart Failure
8. Frailty syndrome and self-care ability in elderly patients with heart failure
9. Frailty and influencing factors in elderly patients with chronic heart failure
10. Frailty and its influencing factors of elderly patients with chronic heart failure
11. A Simple Risk Score Based on Routine Clinical Parameters Can Predict Frailty in Hospitalized Heart Failure Patients
12. Clinical characteristics and frailty status in heart failure with preserved vs. reduced ejection fraction
13. Peak Work Rate during Exercise Could Detect Frailty Status in Elderly Patients with Stable Heart Failure
14. Models to predict prognosis in older patients with heart failure complicated by pre-frailty and frailty: a pilot prospective cohort study
15. Utilizing shared frailty with the Cox proportional hazards regression: Post discharge survival analysis of CHF patients
16. Development of a Diagnostic Model focusing on Nutritional Indicators for Frailty Classification in People with Chronic Heart Failure
17. Analysis of frailty related factors in elderly patients with chronic heart failure

Supplementary Material C: The overall risk of bias for all included studies

Table 1 Quality evaluation of the eligible cohort studies with Newcastle–Ottawa scale

| Study | Patient eligibility | | | | Competences and setting | Rationale | | | Score |
| --- | --- | --- | --- | --- | --- | --- | --- | --- | --- |
|  | Representative-ness | Selection of  non-exposed | Ascertainment  of exposure | Outcome not present at start | Comparability of cohorts on the basis of the design or analysis | Assessment of outcome | Long enough follow-up | Adequacy  (completeness) of follow-up | Total |
| Hamada (2021) | * | * | * | * | * | * | * | * | 9/9 |
| Nozaki (2020) | * | * | * | - | * | * | * | * | 8/9 |
| Noda (2023) | * | * | * | - | * | * | * | * | 8/9 |
| Zhuo (2018) | * | * | * | - | * | * | * | * | 8/9 |
| Tang (2023) | * | * | * | - | * | * | * | * | 8/9 |
| Wang (2023) | * | * | * | - | * | * | * | * | 8/9 |
| *indicates criterion met; - indicates significant of criterion not met. | | | | | | | | | |

Table 2 Quality evaluation of the eligible case-control studies with Newcastle–Ottawa scale.

| Study | Selection | | | | Comparability | Exposure | | Score |
| --- | --- | --- | --- | --- | --- | --- | --- | --- |
|  | Case definition adequate | Representativ-eness of the cases | Selection of controls | Definition of controls | Basis of the  design or analysis | Ascertainment of exposure | Non-response  rate | Total |
| Gao (2022) | * | * | * | * | * | * | - | 6/7 |
| Yang (2021) | * | * | * | * | * | * | - | 6/7 |
| Yang (2022) | * | * | * | * | * | * | - | 6/7 |
| *indicates criterion met; - indicates significant of criterion not met. | | | | | | | |  |

Table 3 Risk of bias assessment for included cross-sectional studies about factors associated with frailty in chronic heart failure

| Study | 1) | 2) | 3) | 4) | 5) | 6) | 7) | 8) | 9) | 10) | 11) | total score |
| --- | --- | --- | --- | --- | --- | --- | --- | --- | --- | --- | --- | --- |
| Valdiviess（2021） | Yes | Yes | Yes | Yes | Yes | Unclear | Yes | Yes | Yes | Yes | Unclear | 9/11 |
| Son(2018) | Yes | Yes | Yes | Yes | Yes | Unclear | No | Yes | Yes | Yes | Unclear | 8/11 |
| Son(2022) | Yes | Yes | Yes | Yes | Yes | Unclear | Yes | No | Unclear | Yes | Unclear | 7/11 |
| Ribeiro(2022) | Yes | Yes | No | Yes | Yes | Yes | No | Yes | Yes | Yes | Unclear | 8/11 |
| Komici (2020) | Yes | Yes | No | Yes | Yes | Yes | Yes | No | Yes | Yes | Unclear | 8/11 |
| Quan (2017) | Yes | Yes | Yes | Yes | Yes | Yes | Yes | No | Yes | Yes | Unclear | 9/11 |
| Wang(2022) | Yes | Yes | Yes | Yes | Yes | No | No | No | Yes | Yes | Unclear | 7/11 |
| Li(2023) | Yes | Yes | Yes | Yes | Yes | Unclear | No | Yes | Yes | Yes | Unclear | 8/11 |
| Tang(2024) | Yes | Yes | Yes | Yes | Yes | Unclear | No | Yes | Yes | Yes | Unclear | 8/11 |
| Song(2023) | Yes | Yes | Yes | Yes | Yes | Unclear | No | Yes | Yes | No | Unclear | 7/11 |
| Tan(2024) | Yes | Yes | Yes | Yes | Yes | Unclear | No | Yes | Yes | No | Unclear | 7/11 |
| She (2023) | Yes | Yes | Yes | Yes | Yes | Unclear | No | Yes | Yes | Yes | Unclear | 8/11 |
| Lv(2024) | Yes | Yes | Yes | Yes | Yes | Unclear | No | Yes | Yes | Yes | Unclear | 8/11 |
| Chen(2022) | Yes | Yes | Yes | Yes | Yes | Yes | No | Yes | Yes | Yes | Unclear | 9/11 |

1) Define the source of information (survey, record review); 2) List inclusion and exclusion criteria for exposed and unexposed subjects (cases and controls) or refer to previous publications; 3)Indicate time period used for identifying patients; 4) Indicate whether or not subjects were consecutive if not population-based; 5)Indicate if evaluators of subjective components of study were masked to other aspects of the status of the participants; 6)Describe any assessments undertaken for quality assurance purposes (e.g., test/retest of primary outcome measurements); 7) Explain any patient exclusions from analysis; 8) Describe how confounding was assessed and/or controlled; 9)If applicable, explain how missing data were handled in the analysis; 10)Summarize patient response rates and completeness of data collection; 11)Clarify what follow-up, if any, was expected and the percentage of patients for which incomplete data or follow-up was obtained

Supplementary Material D: Subgroup analyses of the pooled prevalence of frailty by age(A), study region(B), assessment tools(C), study design(D) and publication year(E).

A


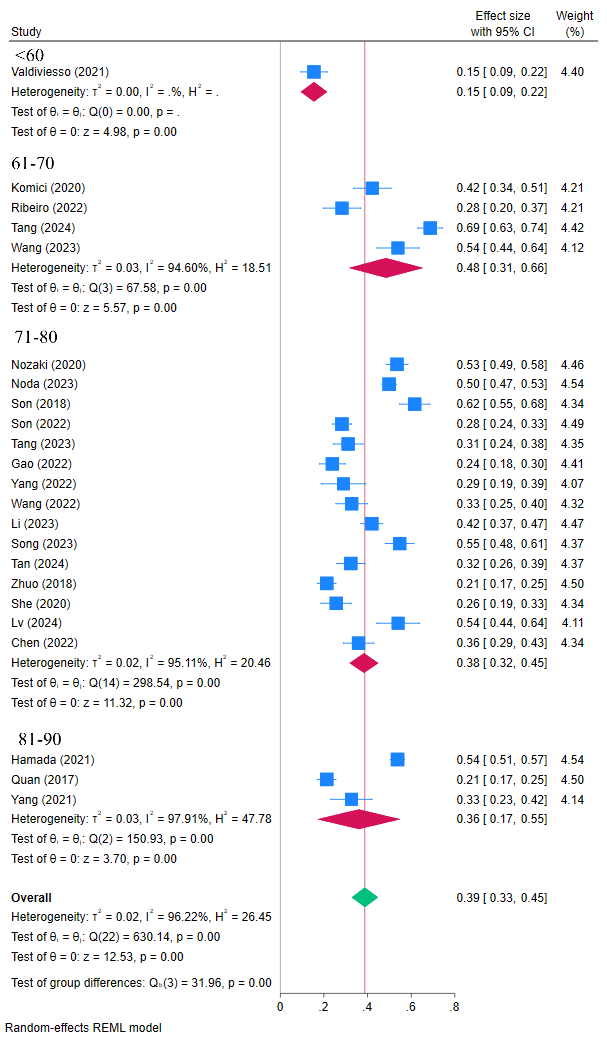


B


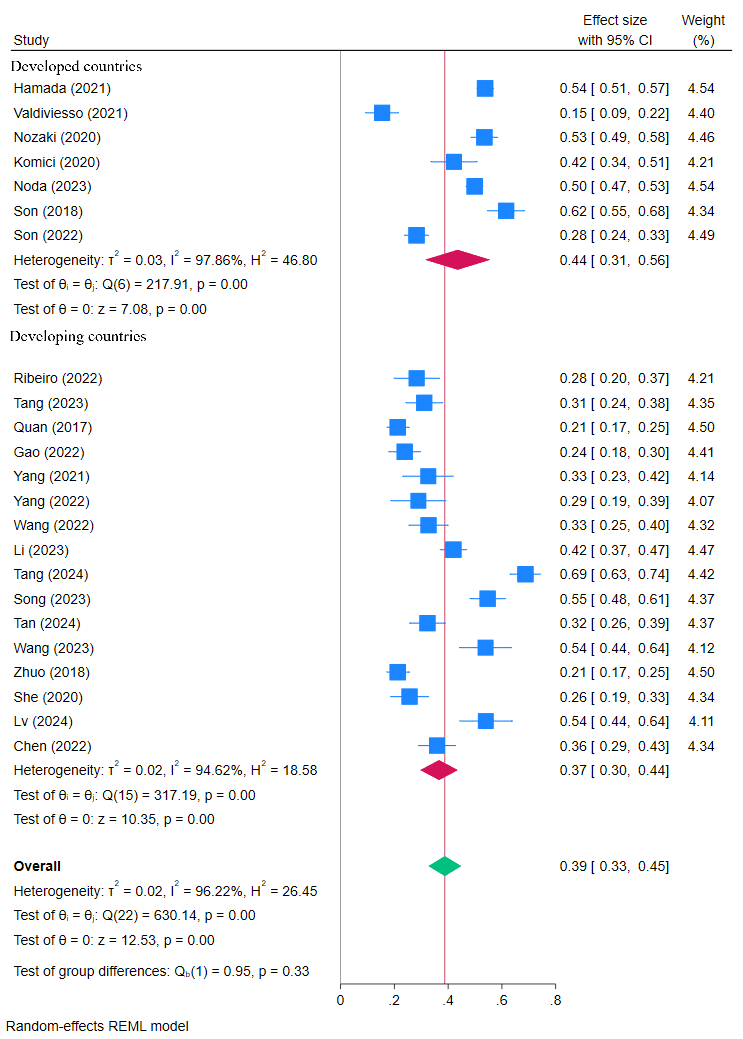


C


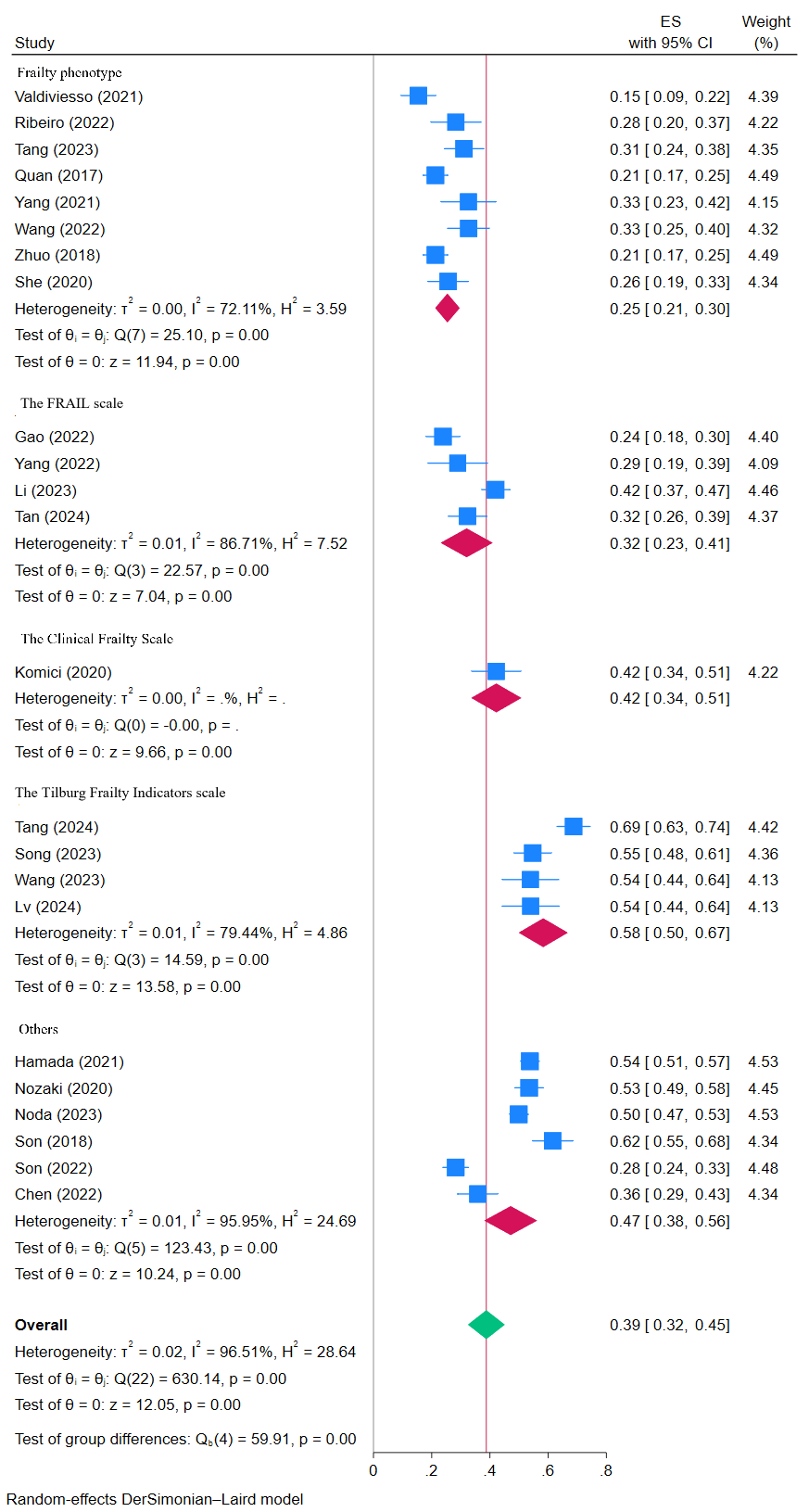


D


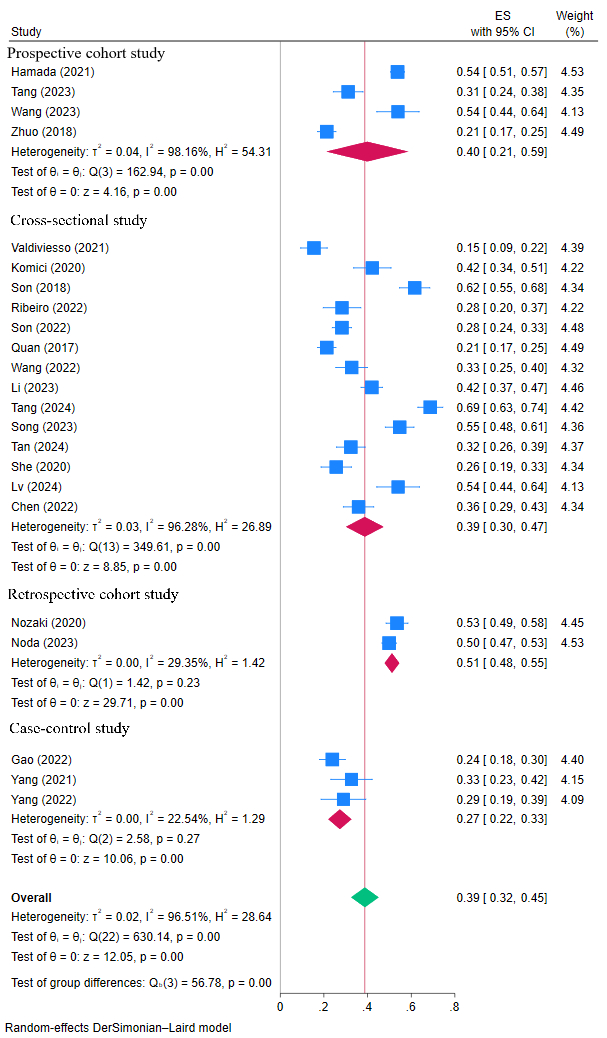


E


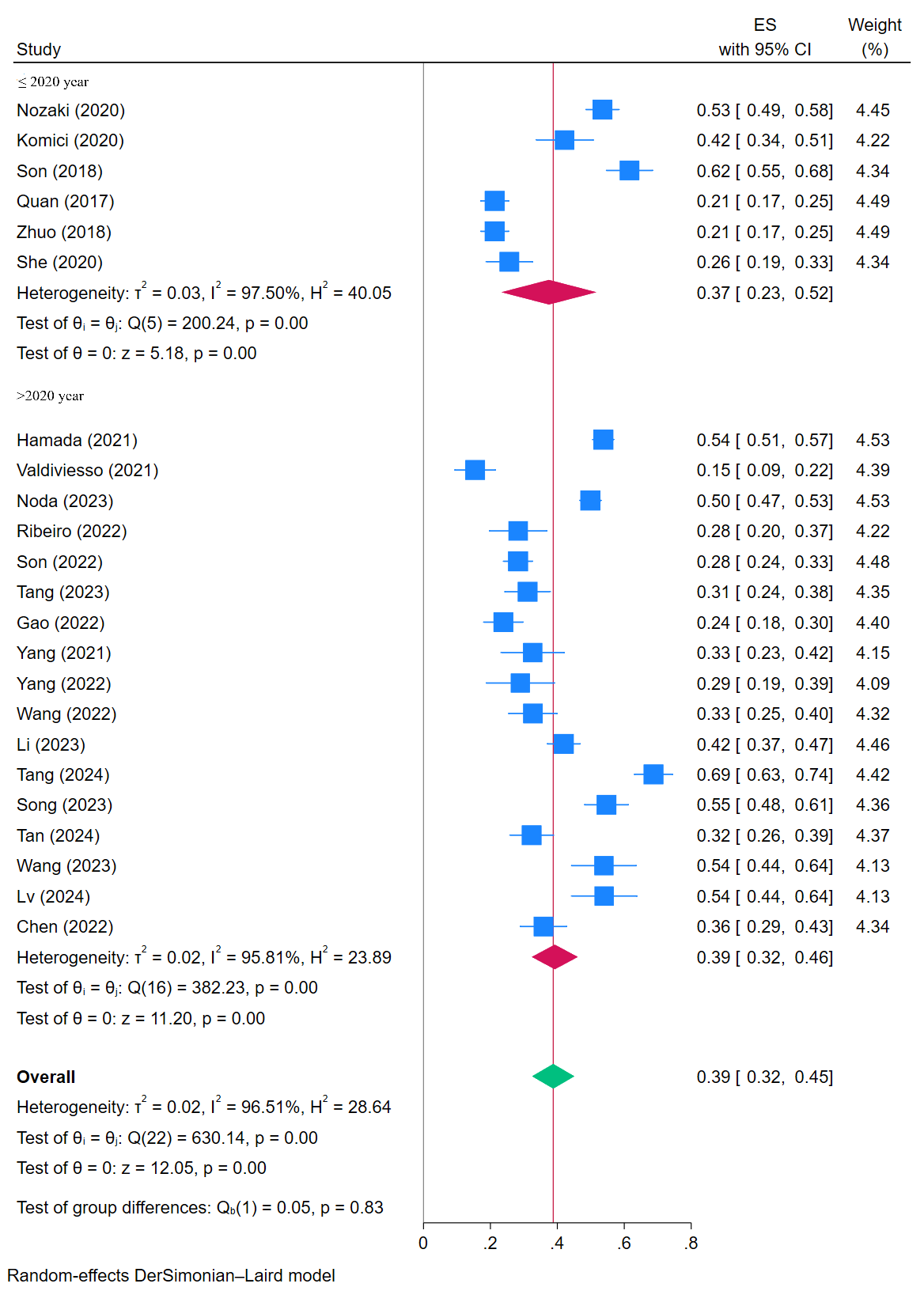


Supplementary Material E: The effect of age(a), albumin(b), cerebrovascular accidents(c), haemoglobin(d), duration of hospitalization(e), left atrial diameter(f), LVEF(g) and number of comorbidities(h) , NYHA functional class(i) and sleep quality(j) on frailty in patients with CHF.

a


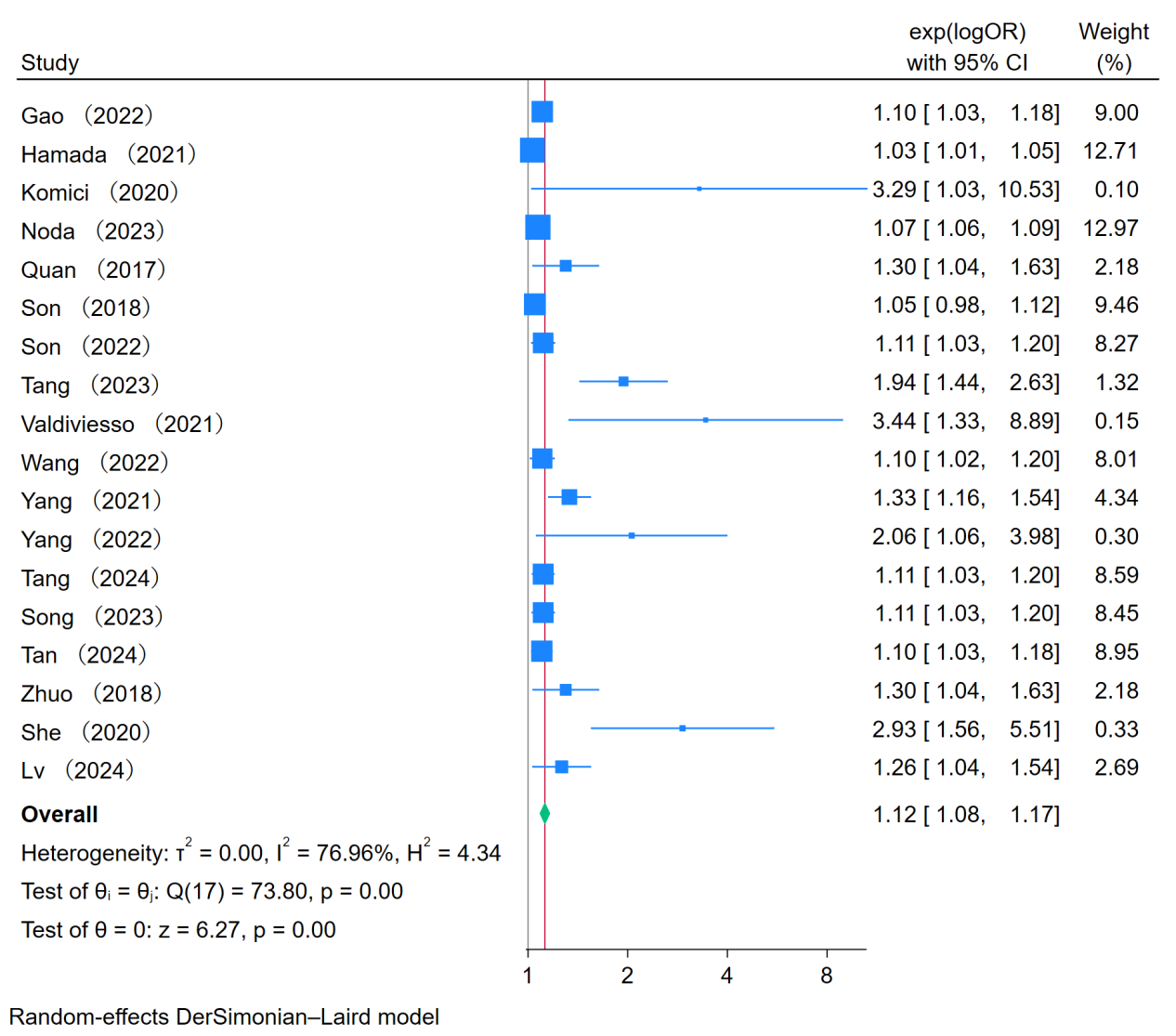


b


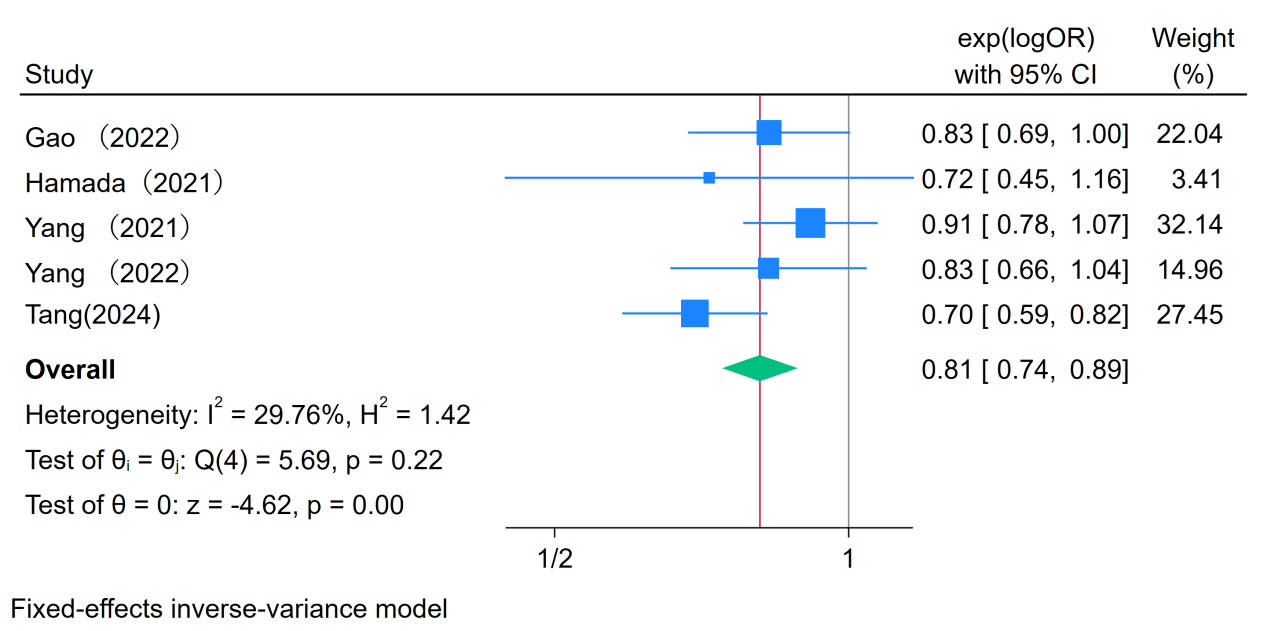


c


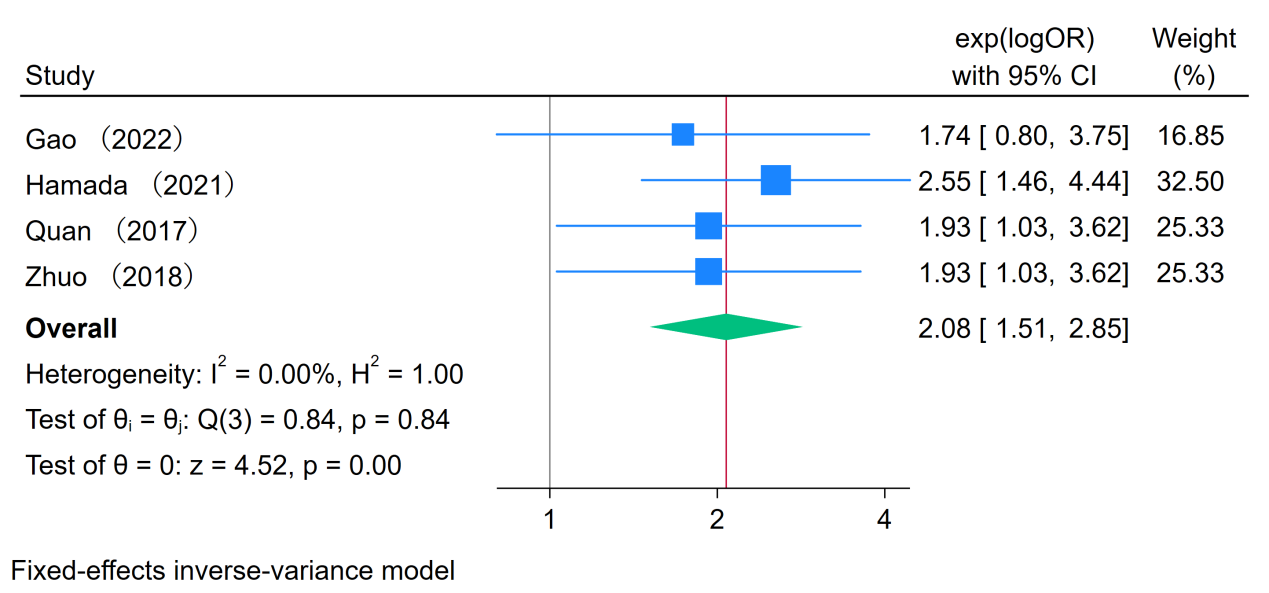


d


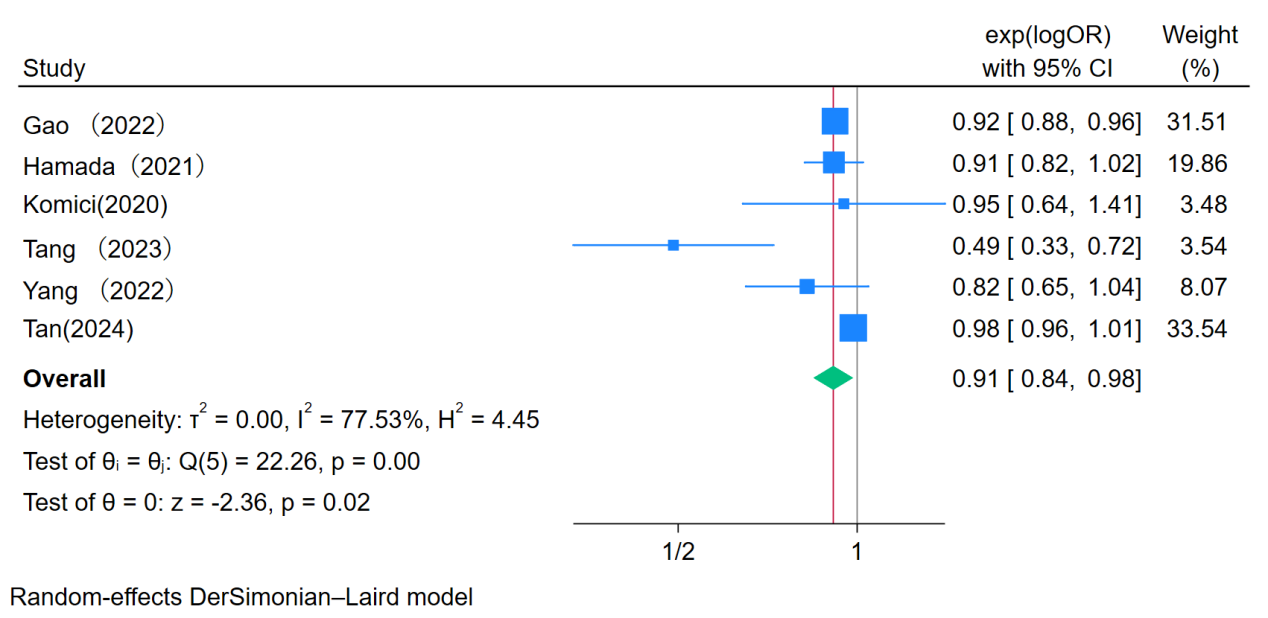


e


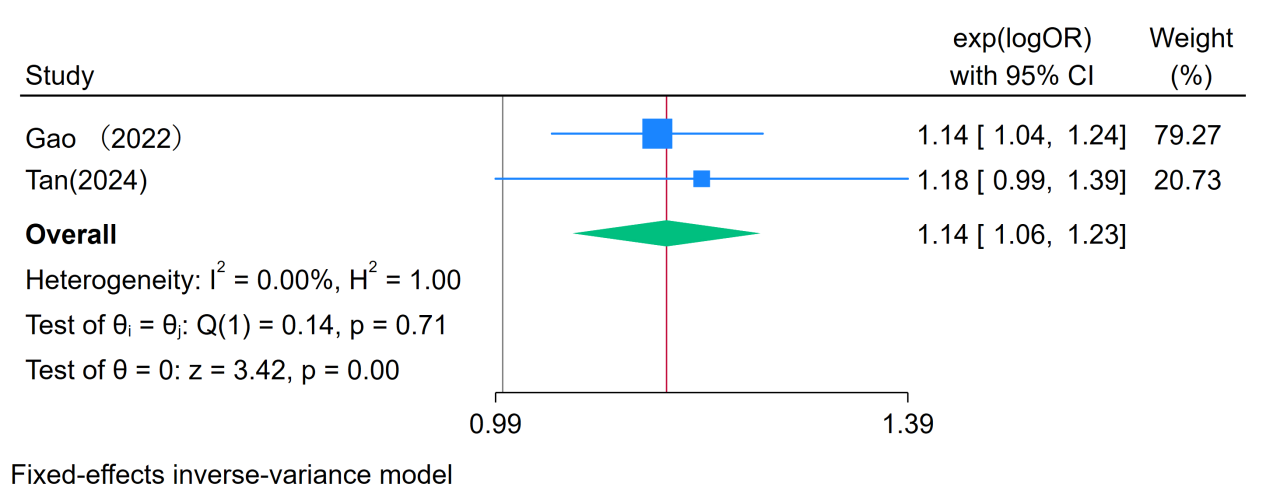


f


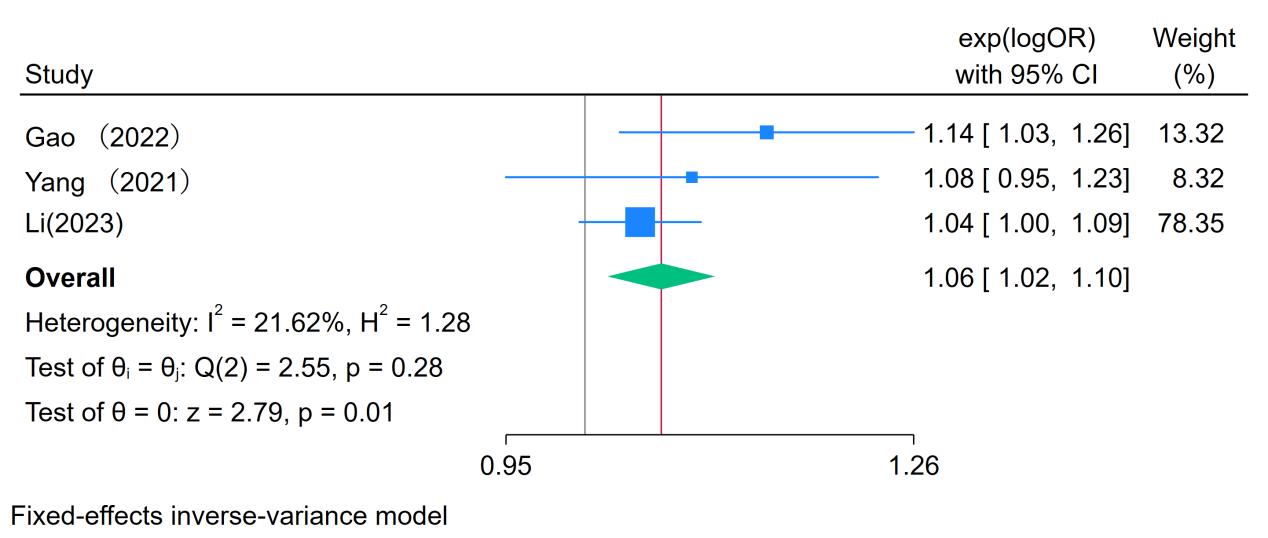


g


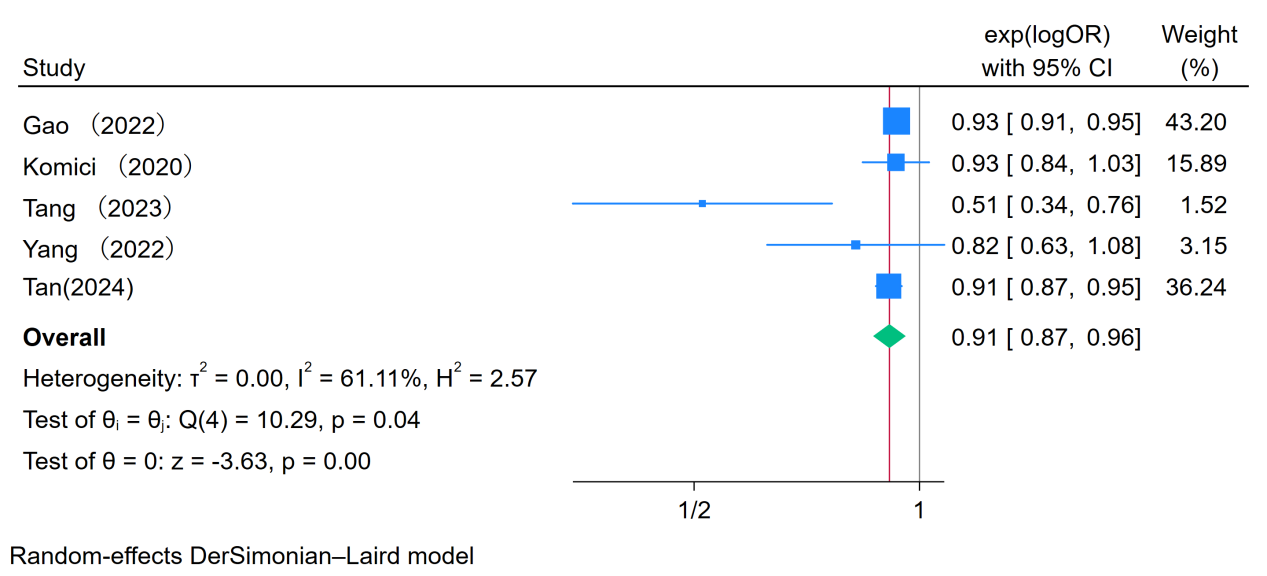


h


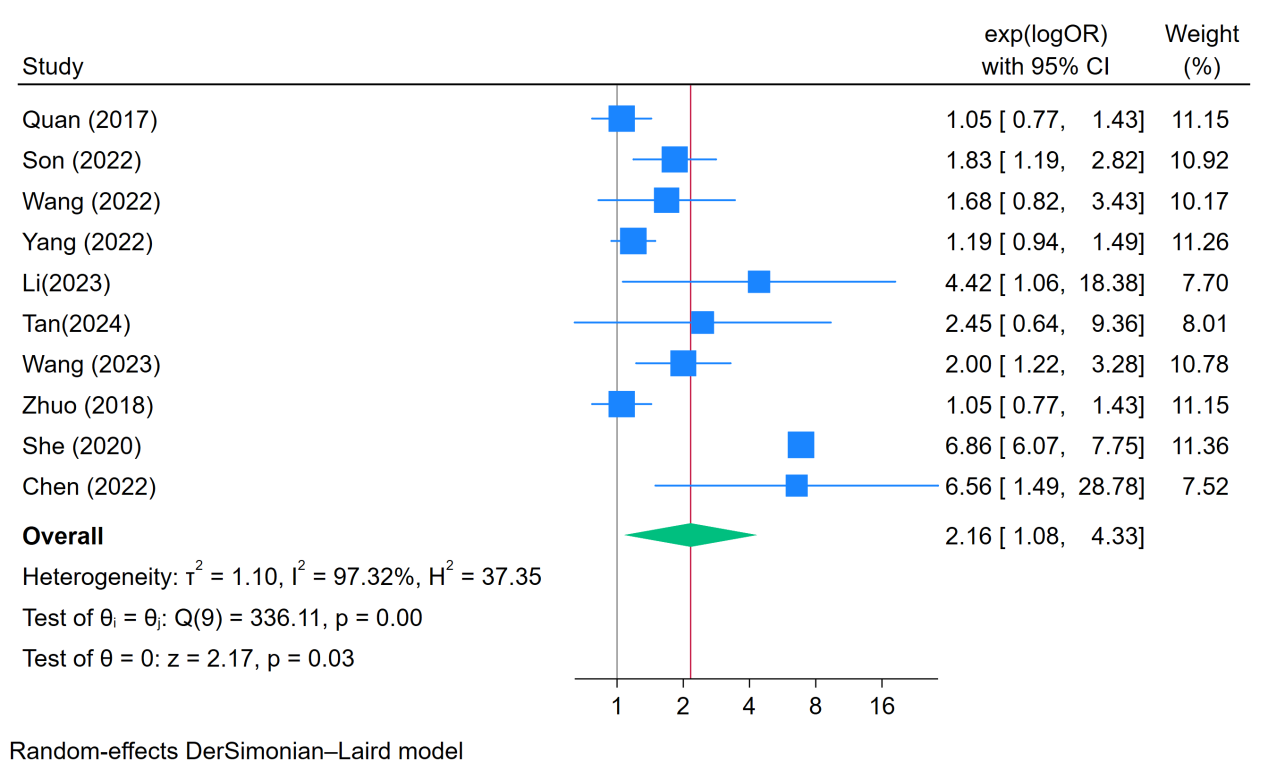


i


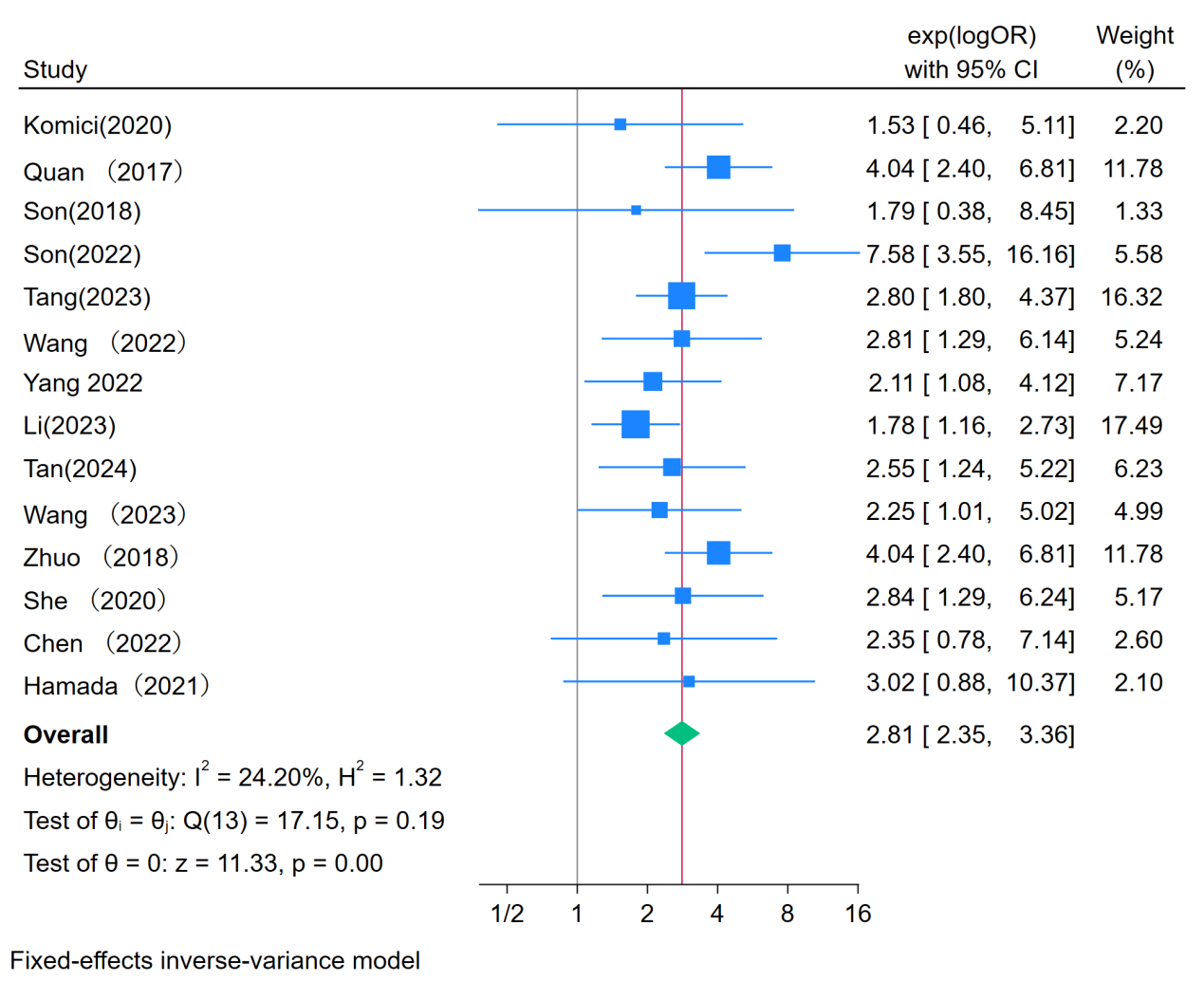


j


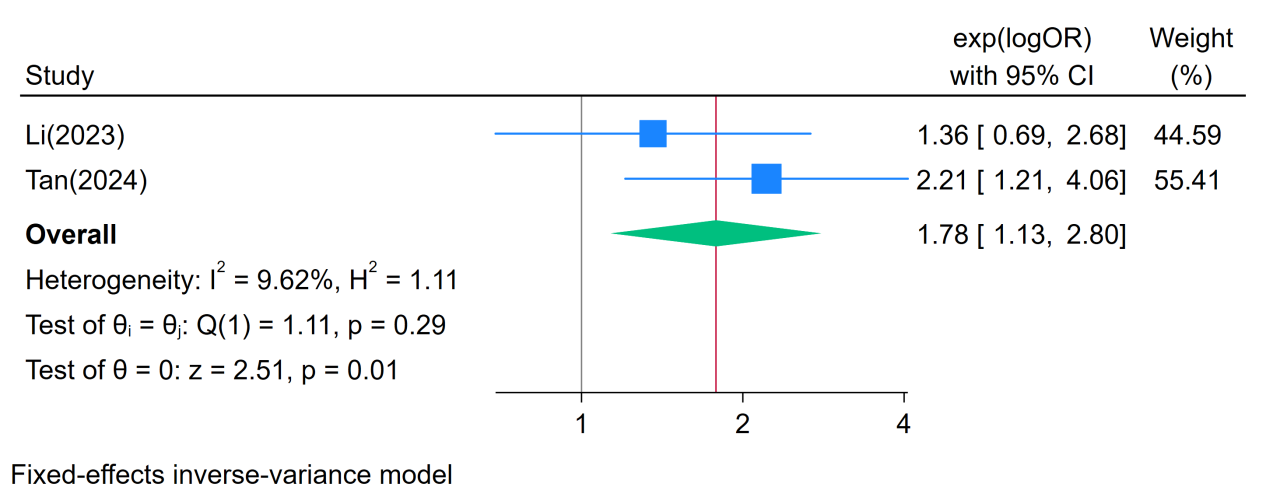


Supplementary Material F: Sensitivity analysis for prevalence of frailty (1) and the effect of age on frailty (2) in patients with CHF.

1


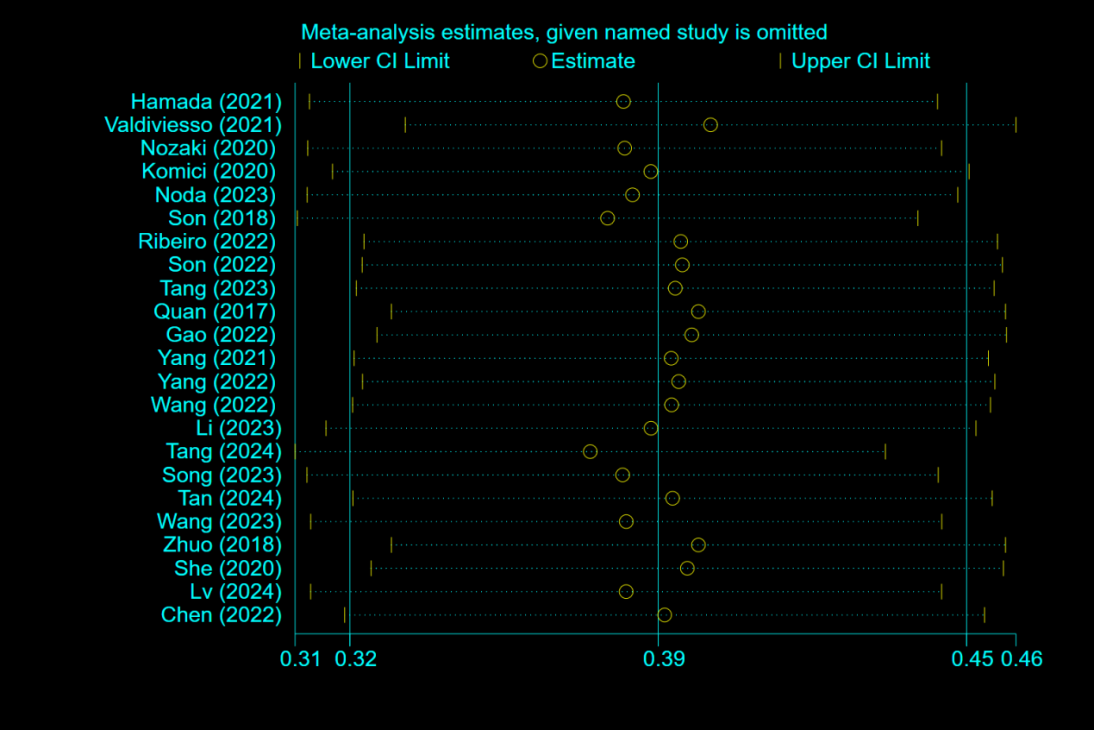


2


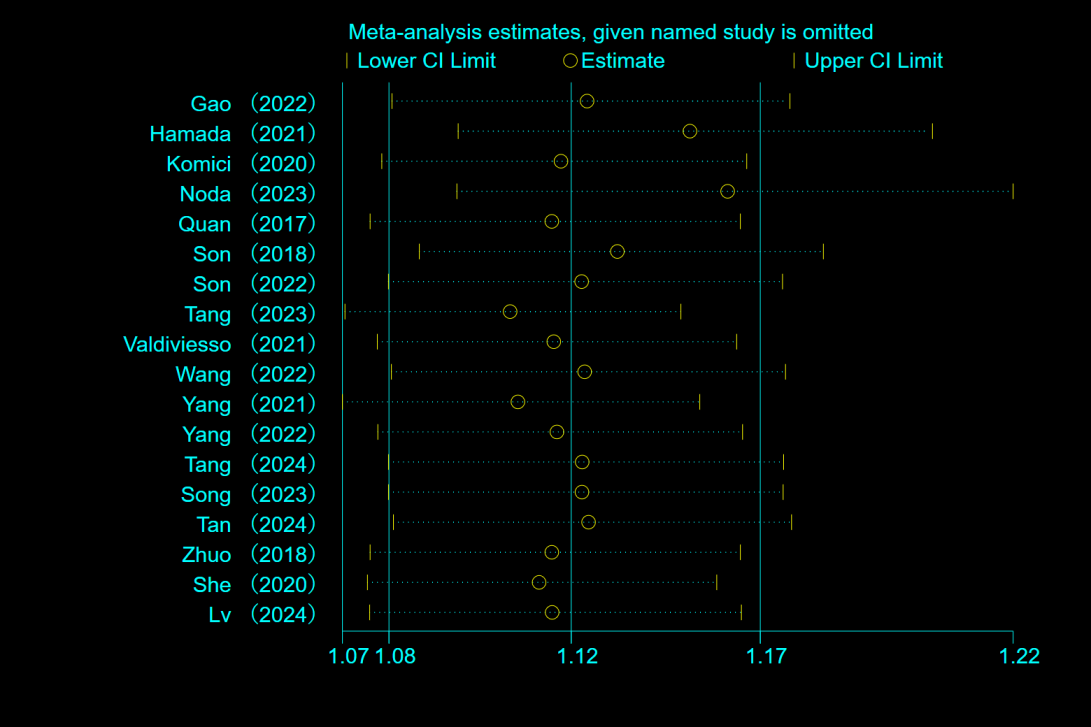


Supplementary Material G: publication bias for prevalence of frailty (3) and the effect of age on frailty (4) in patients with CHF.

3


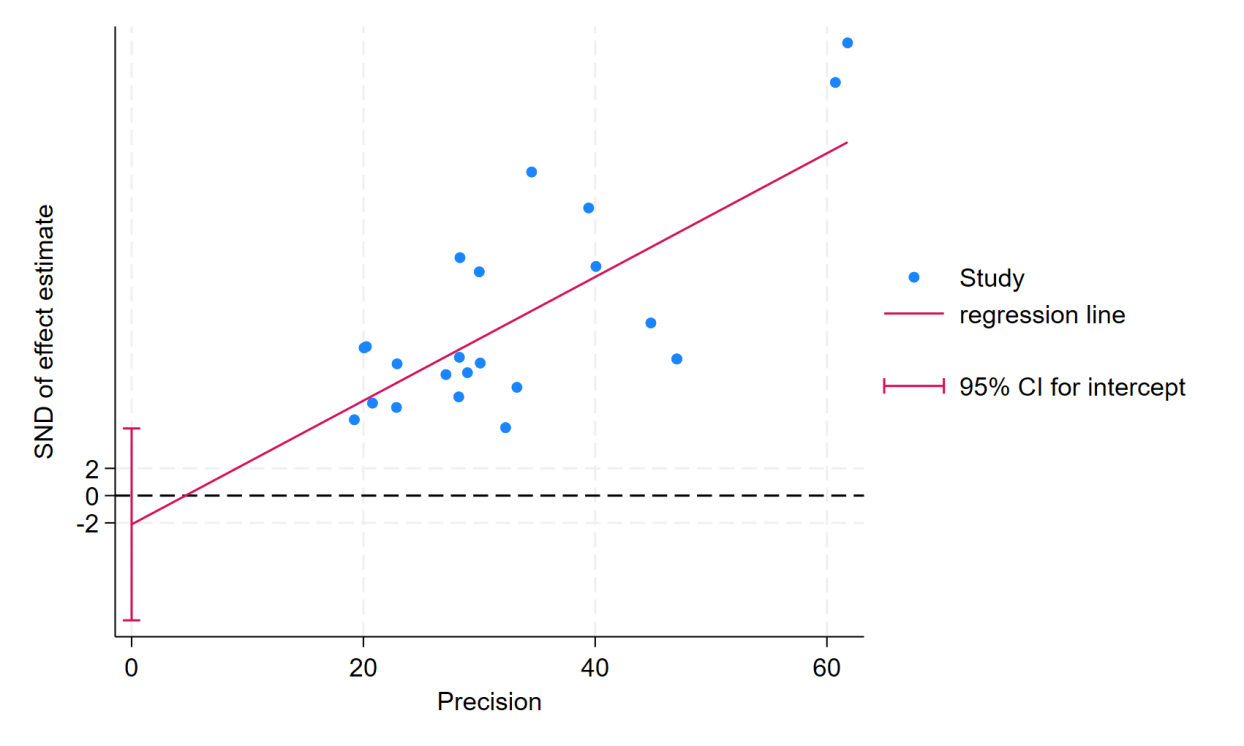


4


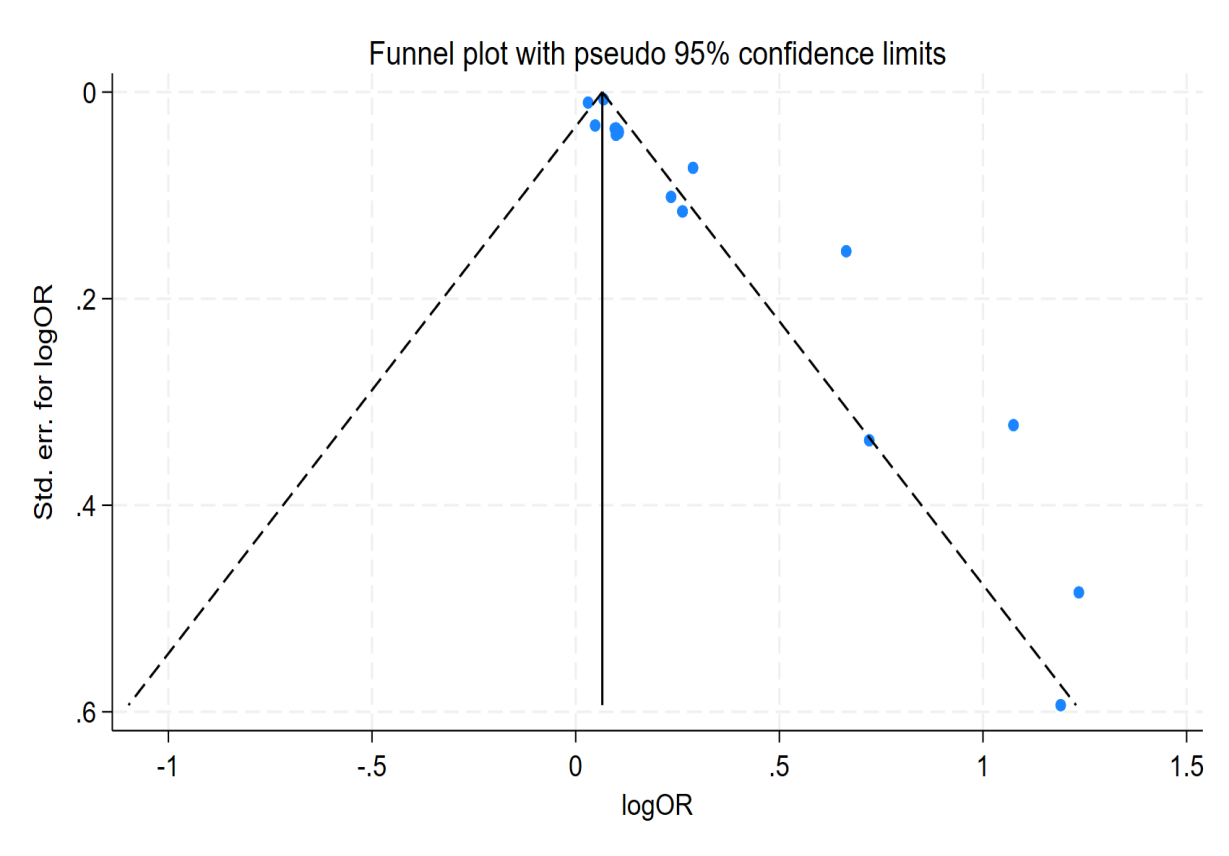

Supplement: Supplementary file 1 [file 2153-8174-26-3-26854-s1.zip › Additional file 3 Supplementary material.docx]
